# Quantitative proteomic analysis of prostate tissue specimens identifies deregulated protein complexes in primary prostate cancer

**DOI:** 10.1186/s12014-019-9236-2

**Published:** 2019-04-13

**Authors:** Bo Zhou, Yiwu Yan, Yang Wang, Sungyong You, Michael R. Freeman, Wei Yang

**Affiliations:** Division of Cancer Biology and Therapeutics, Departments of Surgery and Biomedical Sciences, Samuel Oschin Comprehensive Cancer Institute, Cedars-Sinai Medical Center, Rm. 4009, Davis Research Bldg 8700 Beverly Blvd, Los Angeles, CA 90048 USA

**Keywords:** Differential co-regulation analysis, Differential expression analysis, Prostate cancer, Protein complex, Quantitative proteomics, TMT-SPS-MS3, Tissue

## Abstract

**Background:**

Prostate cancer (PCa) is the most frequently diagnosed non-skin cancer and a leading cause of mortality among males in developed countries. However, our understanding of the global changes of protein complexes within PCa tissue specimens remains very limited, although it has been well recognized that protein complexes carry out essentially all major processes in living organisms and that their deregulation drives the pathogenesis and progression of various diseases.

**Methods:**

By coupling tandem mass tagging-synchronous precursor selection-mass spectrometry/mass spectrometry/mass spectrometry with differential expression and co-regulation analyses, the present study compared the differences between protein complexes in normal prostate, low-grade PCa, and high-grade PCa tissue specimens.

**Results:**

Globally, a large downregulated putative protein–protein interaction (PPI) network was detected in both low-grade and high-grade PCa, yet a large upregulated putative PPI network was only detected in high-grade but not low-grade PCa, compared with normal controls. To identify specific protein complexes that are deregulated in PCa, quantified proteins were mapped to protein complexes in CORUM (v3.0), a high-quality collection of 4274 experimentally verified mammalian protein complexes. Differential expression and gene ontology (GO) enrichment analyses suggested that 13 integrin complexes involved in cell adhesion were significantly downregulated in both low- and high-grade PCa compared with normal prostate, and that four Prothymosin alpha (ProTα) complexes were significantly upregulated in high-grade PCa compared with normal prostate. Moreover, differential co-regulation and GO enrichment analyses indicated that the assembly levels of six protein complexes involved in RNA splicing were significantly increased in low-grade PCa, and those of four subcomplexes of mitochondrial complex I were significantly increased in high-grade PCa, compared with normal prostate.

**Conclusions:**

In summary, to the best of our knowledge, the study represents the first large-scale and quantitative, albeit indirect, comparison of individual protein complexes in human PCa tissue specimens. It may serve as a useful resource for better understanding the deregulation of protein complexes in primary PCa.

**Electronic supplementary material:**

The online version of this article (10.1186/s12014-019-9236-2) contains supplementary material, which is available to authorized users.

## Background

Prostate cancer (PCa) is the most frequently diagnosed non-skin cancer and a leading cause of cancer death among males in developed countries [1]. In the United States alone, it was estimated that 174,650 men will be diagnosed with PCa and that 31,620 will die of this disease in 2019 [2]. Largely owing to the developments and advances in next-generation sequencing technologies, the past few years have witnessed a striking growth of genomic and transcriptomic profiles of clinical PCa specimens [3]. These large-scale efforts not only enhanced our understanding of the molecular underpinnings of PCa pathogenesis and progression, but also facilitated the identification of novel PCa biomarkers and therapeutic targets [4, 5]. Nonetheless, despite the significant progress, genomic and transcriptomic profiling studies have inherent limitations—they only indirectly and often inconclusively measure the properties of proteins, which are the major functional molecules and actual executors of biological functions in living organisms. In fact, although genomic alterations can be prioritized using a systems pharmacology approach combined with targeted proteomics [6], recent studies have suggested that aberrations at the gene copy number, DNA methylation, and RNA expression levels often do not reliably predict changes at the protein expression level [7, 8].

In contrast to genomic and transcriptomic technologies, mass spectrometry (MS)-based proteomic technologies enable comprehensive and direct analysis of proteins, and have thus been widely used in the proteomic profiling of clinical specimens, such as biofluid and tissue samples [9]. Compared with biofluid specimens such as blood and urine, tissue specimens allow more accurate sampling of proteomic changes in tumor cells and microenvironment, but they are more difficult to obtain. According to a recent survey, only about 40 proteomic studies were performed on human PCa tissue specimens in the past decade [10]. Moreover, most of these studies were conducted using the two-dimensional electrophoresis (2DE) matrix-assisted laser desorption ionization mass spectrometry (MALDI-MS) technology, which rarely provides adequate proteomic coverage and is only semi-quantitative. To date, comprehensive and quantitative proteomic studies of PCa tissue specimens have remained scarce [7, 11–15]. Furthermore, none of these studies investigated the global changes of multiprotein complexes along PCa development and progression. Notably, protein complexes act as highly specialized molecular machines and carry out essentially all major processes in a cell, such as gene transcription and splicing as well as protein synthesis and degradation [16, 17]. The abnormal expression and/or activation of certain protein complexes may lead to the pathogenesis and progression of many diseases [18]. Hence, the identification of deregulated protein complexes in clinical tissue specimens offers a great potential of revealing novel molecular mechanisms and discovering new biomarkers and therapeutic targets for various human diseases including PCa.

Currently, a variety of proteomic technologies are available for large-scale protein quantification [19]. Among these, tandem mass tagging (TMT) offers high multiplexing capability, allowing quantitative comparison of up to 11 samples simultaneously [20, 21]. Previously, TMT suffered from the issue of precursor ion interference, which results in ratio compression and thus an underestimation of expression differences [22]. With the recent development of the synchronous precursor selection (SPS)-MS3 technique, the ratio compression issue is largely eliminated [23]. As such, the TMT-SPS-MS3 combination enables highly multiplexed and accurate quantification of proteomes. Moreover, when coupled with protein co-regulation analysis, TMT-SPS-MS3 analysis permits systems-wide analysis of protein–protein interactions (PPIs) with high accuracy [24].

In the present study, the TMT-SPS-MS3 approach was integrated with differential expression and co-regulation analyses to investigate the global changes of protein complexes, in terms of abundance and PPI, in 27 optimal cutting temperature (OCT) compound-embedded and cryopreserved clinical tissue specimens of primary PCa (i.e., 9 normal prostate, 9 low-grade/low-risk PCa, and 9 high-grade/high-risk PCa). Notably, recent studies have shown that, when properly handled and processed, OCT samples provide better protein recovery and MS identification than formalin-fixed and paraffin-embedded (FFPE) specimens [25, 26]. After stringent statistical analysis, the study revealed that certain protein complexes were significantly deregulated in low-grade and/or high-grade PCa, compared with normal prostate. Further exploitation of the deregulated protein complexes may shed new light on the molecular basis of PCa development and progression in vivo, as well as provide novel biomarkers and therapeutic targets for better management of this leading male cancer.

## Methods

### Prostate tissue specimens

All 18 PCa tissue samples and 9 PCa-adjacent normal control samples (with ≤ 2% tumor) were collected from radical prostatectomy during the period of 2010–2014, OCT embedded by the Cedars-Sinai Medical Center Biobank core facility, and stored at − 80 °C (Additional file 2: Table S1). The PCa samples are either of low-grade (Gleason score of 6) or high-grade (Gleason score of 8 or 9) prostate adenocarcinoma (Additional file 2: Table S1). The average (± standard deviation; SD) ages of patients in the normal (N) prostate, low-grade (LG) PCa, and high-grade (HG) PCa groups are 62.1 (± 11.2), 61.3 (± 9.2), and 65.1 (± 8.1) years, respectively (Additional file 2: Table S1). Some patients had comorbidities such as hypertension and arthritis, but there is no significant bias for a specific type of comorbidity in any group (Additional file 2: Table S1).

To estimate the percentages of epithelial and stromal cells in each tissue specimen, cryostat sections were cut from OCT blocks at 8 μm with a Leica CM1950 Cryostat (Leica Biosystems, Wetzlar, Germany) and mount on gelatin-coated histological slides. After hematoxylin and eosin (H and E) staining, tissue sections were evaluated by an experienced pathologist, who was blinded to the experimental design, to estimate the percentages of epithelial and stromal cells under an optical microscope. Only specimens with ≥ 60% of epithelial cell purity were used for the proteomic analysis (Additional file 2: Table S1).

### Protein extraction, digestion and TMT labeling

OCT was removed essentially as described [27]. Briefly, with a sterile scalpel, about 20 mm^3^ tissue was cross-sectionally cut from the top (tissue) layer of each OCT block in a petri dish on ice, further cut into small pieces, and transferred to a 1.5 mL Eppendorf tube. Tissue pieces were gently washed with 1 mL ice-cold 70% ethanol for twice, ice-cold water for once, and ice-cold 100 mM Tris–HCl, pH 7.4 for twice. To lyse tissue, 100 μL lysis buffer (80 mM Tris–HCl, 4% SDS, 100 mM DTT, pH7.4) was added into each tube, and the tissue pieces were grinded with disposable pestles using a cordless pestle motor (VWR, Radnor, PA). The lysates were thoroughly sonicated in a water-bath sonicator (Elma S180H) to reduce viscosity, incubated at 95 °C for 5 min, and centrifuged at 16,000 × g for 10 min. Protein concentration was determined using the Pierce 660 nm protein assay (Thermo Scientific) according to the manufacture’s instruction.

To generate an internal proteomic standard, 20 μg protein from each of the 27 samples was mixed. Because a 10-plex TMT reagent set can only accommodate up to 10 samples, the 27 tissue samples and three internal standard (pooled) samples were divided into three sets. Each TMT10plex set contains one internal standard, three normal prostate, three low-grade PCa, and three high-grade PCa samples. From each sample, 60 μg proteins were alkylated with iodoacetamide and digested with trypsin using the filter-aided sample preparation (FASP) method [27]. Tryptic peptides were labeled with 10-plex TMT reagents in parallel, essentially as we previously described [28, 29]. To ensure that the internal standards for the three sets are identical, the three TMT126-labeled internal standard samples were mixed into one single sample. Subsequently, for each TMT10plex set, an equal amount of tryptic peptides (derived from about 20 μg proteins) with differential TMT labeling was merged into one sample, desalted using C_18_ spin columns (Thermo Scientific), and concentrated in a SpeedVac (Thermo Scientific).

### Peptide fractionation

To reduce the complexity of tryptic peptides and improve the proteomic coverage, peptide fractionation was performed using high-pH reversed-phase liquid chromatography (LC) [30]. Each TMT10plex-labeled peptide mixture sample was redissolved with 45 μL 10 mM ammonium formate, pH 10. Twenty microliters of peptide solution were injected and separated on a 20-cm Hypersil GOLD C_18_ column (1.9 μm particle size, 2.1 mm inner diameter, 175 Å pore size) heated to 35 °C on an Ultimate 3000 XRS system (Thermo Scientific), with a flow rate of 0.5 mL/min. Mobile phase A and B consisted of 10 mM ammonium formate in water (pH 10) and 10 mM ammonium formate in 95% acetonitrile (pH 10), respectively. The 13-min LC gradient was 0% B over 3 min, 0–28% B over 7 min, 28–90% B over 1 min, 90% B over 1 min, and 90–0% B over 1 min. For each TMT10plex set, a total of 72 fractions were collected after 3.5 min, with a collection rate of one fraction per 6 s. The 72 fractions were then concatenated into 24 fractions by combining fractions 1, 25, 49; 2, 26, 50; and so on. It was shown that the concatenation strategy allows more uniform peptide distributions on subsequent low-pH RPLC and thus improves protein identifications [30]. The concatenated fractions were concentrated in a SpeedVac and stored at -80 °C until LC-SPS-MS3 analysis.

### LC-SPS-MS3 analysis

LC-SPS-MS3 analysis was conducted on an EASY nLC 1200 connected to an Orbitrap Fusion Lumos mass spectrometer (Thermo Scientific). The Orbitrap Fusion Lumos is currently one of the most advanced mass spectrometers. It is a tribrid mass spectrometer that contains three different types of mass analyzers—a quadrupole mass filter, a linear ion trap, and an orbitrap. The tribrid configuration allows isolation and MS3 fragmentation of multiple MS2 fragment ions (i.e., SPS-MS3), largely eliminating the issues of isolation interference and dynamic range compression that are commonly observed in isobaric tag-based quantitative proteomics experiments [23].

Each fraction of peptides was redissolved with 25 μL 0.2% formic acid, 2% acetonitrile. Ten microliters of peptide solution were loaded onto a 2-cm trap column (PepMap 100 C_18_, 75 μm inner diameter, 3 μm particles, 100 Å pore size) and separated by a 50-cm EASY-Spray column (PepMap RSLC C_18_, 75 μm inner diameter, 2 μm particles, 100 Å pore size) heated to 55 °C, at a flow rate of 250 nL/min. Mobile phases A and B consisted of 0.1% formic acid in water and 0.1% formic acid in 80% acetonitrile, respectively. The 3-h LC gradient was 3–25% B over 140 min, 25–50% B over 25 min, 50–100% B over 5 min, and 100% B over 10 min. SPS-MS3 analysis was conducted essentially as described [31]. The parameter settings for FTMS1 include orbitrap resolution (120,000), scan range (350–1400), AGC (5E5), maximum injection time (100 ms), RF lens (30%), data type (centroid), charge state (2–5), dynamic exclusion for 60 s using a mass tolerance of 7 ppm, and internal calibration using *m/z* 371.10123; for ITMS2 include mass range (400–1400), number of dependent scans (10), isolation window (0.4 *m/z*), activation type (rapid CID), collision energy (35%), maximum injection time (120 ms), AGC (2E4), and data type (centroid); for MS3 include mass range (400–1400), precursor ion exclusion (low *m/z* 50, high *m/z* 5), isolation window (*m/z* 0.7), MS2 isolation window (*m/z* 2), number of notches (10), HCD collision energy (55%), orbitrap resolution (50,000), maximum injection time (150 ms), AGC (2.5E5), and data type (centroid).

### Protein Identification and Quantification

To determine TMT labeling efficiency, RAW files for three fractions (#4, #12, #20) were selected from each TMT10plex set and analyzed by Proteome Discoverer (v2.2) (Thermo Scientific). The SEQUEST HT algorithm was applied to search against the human Uniprot protein sequence database (released on 03/30/2018, containing 20,937 canonical sequences and 72,379 additional sequences). Of note, the 72,379 additional sequences are all human proteins, including ~ 22,000 manually reviewed non-canonical isoform sequences as well as ~ 50,000 additional predicted and unreviewed sequences in Uniprot/TrEMBL. Modifications included carbamidomethylation of cysteines as fixed modification as well as acetylation of protein N-terminus, oxidation of methionines, TMT tagging of lysines and peptide N-terminus as variable modifications. Trypsin (Full) was used for digestion and up to two mis-cleavages were allowed. The mass tolerance was 10 ppm for precursor mass and 0.6 Da for fragment mass. Peptide-spectrum matches (PSMs) identified with high confidence and unambiguously were used to determine labeling efficiency, by calculating the ratios of PSMs for TMT-labeled peptides against total PSMs.

To identify and quantify proteins, all the 72 acquired LC-SPS-MS3 files (24 files per TMT10plex set × 3 sets) were analyzed by MaxQuant (v1.6.0.16) [32], using the Andromeda algorithm [33] to search against the aforementioned human Uniprot protein sequence database combined with the common contaminant protein sequences (244 sequences). The quantification type was Reporter ion MS3, the isobaric labels were TMT10plex, and the reporter mass tolerance was 0.003 Da. Modifications included carbamidomethylation of cysteines as fixed modification as well as acetylation of protein N-terminus, deamidation of asparagines and glutamines, and oxidation of methionines and prolines as variable modifications. Tryspin/P was used for digestion and up to two mis-cleavages were allowed. The match-between-runs function was enabled, using 0.7 min of match time window and 20 min of alignment time window. The mass tolerance was 20 ppm for first search peptide tolerance and 4.5 ppm for main search peptide tolerance, and 0.5 Da for MS/MS match tolerance. A false discovery rate (FDR) of 1% was applied to filter PSMs, peptides, and protein groups. The mass spectrometry proteomics data have been deposited to the ProteomeXchange Consortium (http://proteomexchange.org) via the PRIDE partner repository [34] with database identifier PXD010744.

### Identification of differentially expressed proteins (DEPs)

Statistical analysis was performed with Perseus (v1.5.5.3) [35]. Proteins identified from the reverse sequence database or based on a single modified peptide, as well as non-human contaminant proteins identified from the contaminant sequence database, were filtered out. Subsequently, only proteins quantified across all the analyzed samples were selected for statistical analysis. Protein ratios against the internal standard (TMT126 channel) were computed and then log_2_-transformed. For each sample, the log_2_-transformed ratios were normalized against the Tukey’s bi-weight mean, which calculates a robust average that is unaffected by outliers, with the assumption that most identified cellular proteins are not significantly differentially expressed across the samples. To estimate the epithelial/stromal cell purity in the tissue sections used for proteomic analysis, the protein expression data were analyzed using the Estimation of Stromal and Immune Cells in Malignant Tumors using Expression data (ESTIMATE) (v2.0) [36], an algorithm widely used to compute cell purity from expression data.

After a quality control analysis using SuperHirn [37], one outlier sample was detected from each group and they were removed. For the comparison between each group (n = 8 after the removal of outlier samples), Student’s *t* test (two-tailed) was used. To correct the *p* values for multiple testing, the Storey method was applied [38]. DEPs were identified using *q* values < 0.05 and the empirical cutoff of log_2_-transformed fold changes of > 0.5 in absolute value. It is known that stromal content in PCa tissue samples is generally lower than in normal prostate. Thus, some unchanged stromal cell-enriched proteins may appear to be downregulated in PCa tissue compared with normal prostate, and are thus erroneously identified as downregulated DEPs. To determine to what extent DEPs are stromal cell-enriched, we generated a PCa stromal gene signature and compared the signature genes with the downregulated DEPs. To generate the PCa stromal signature, the gene expression profiles for 12 stromal and 89 epithelial cell populations isolated from PCa tissues by laser capture microdissection (GSE6099) [39] were compared, and the cutoffs of false discovery rate (FDR) < 1% and log_2_-transformed fold change > 1 were applied to identify genes significantly enriched in PCa stromal cells compared with epithelial cells. Moreover, the PCa stromal signature was corroborated by analyzing the gene expression data of the GSE8218 cohorts, for which the stromal content ratios were evaluated by a pathologist and provided in [40].

For gene ontology (GO) enrichment analysis of DEPs, the Database for Annotation, Visualization and Integrated Discovery (DAVID, v6.8) analysis was performed [41]. To generate putative PPI networks from DEPs, the Ingenuity Pathway Analysis (IPA) (Ingenuity) was performed with high stringency—only direct PPIs with experimental evidence were used. Subcellular localizations of PPI subnetworks were displayed using the “Subcellular Layout” function of the IPA, which classifies proteins into five subcellular compartments: (1) extracellular space, (2) plasma membrane, (3) cytoplasm, (4) nucleus, and (5) unknown.

### Identification of differentially expressed protein complexes

Proteins quantified across all samples were mapped into individual protein complexes in the CORUM (v3.0) database [42], using the R statistical software (R Development Core Team; https://www.r-project.org/) (v3.5.0). Briefly, the allComplexes.txt and uniprot_corum_mapping.txt files were downloaded from the CORUM database (http://mips.helmholtz-muenchen.de/corum/#download). The protein IDs of quantified proteins (Additional file 2: Table S4) were matched with the UniProtKB accession number in the uniprot_corum_mapping.txt file, with which a CORUM complex ID is associated. The number of quantified subunits in each CORUM complex was counted, and protein complexes with < 2 quantified subunits were removed. For the remaining CORUM complexes, if the number of quantified subunits accounts for < 50% of the number of total subunits (i.e., subunit coverage < 50%), they were removed so as to reduce potentially erroneous quantification at the protein complex level. Furthermore, if two or more different CORUM complexes contain an identical set of quantified subunits, the redundancy was removed as follows: 1) if the subunit coverages were different, only the CORUM complex with the highest subunit coverage was kept, and 2) if the subunit coverages were identical, only the CORUM complex with the smallest CORUM ID number was kept.

To identify specific protein complexes that are differentially expressed, the mean log_2_-ratio of all proteins in a CORUM complex was calculated for each sample, and then compared across the three groups (i.e., normal, low-grade, and high-grade) by Student’s *t*-test (two-tailed). The Storey method was then applied to correct the *p* values for multiple testing. The CORUM complexes with *q* values of < 0.05 and the mean difference of > 0.31 in absolute value were accepted as differentially expressed complexes. Here, the cutoff for the mean difference was set as 0.31 because it corresponds to *p* < 0.05, based on the normal distribution of mean differences (SD = 0.158). To identify significantly enriched GO terms, Fisher exact test was performed by using Perseus (v1.5.5.3) [35] and the cutoffs of *q* < 0.05 and enrichment factor > 2 were applied.

### Identification of differentially regulated protein complexes

Differential co-regulation analysis provides a level of information about PPIs, which is not possible to obtain using the widely used differential expression analysis [24, 43]. Prior to the co-regulation analysis, the CORUM complexes used for the aforementioned differential expression analysis were further filtered to remove those with PPI coverages of < 50%. Here, the PPI coverage for a protein complex is defined by the number of inter-subunit PPIs (i.e., excluding self-pairs) for quantified subunits divided by the number of inter-subunit PPIs for all subunits. For a protein complex containing n subunits, the number of inter-subunit PPIs is n × (n − 1)/2.

Differential co-regulation analysis was conducted using R (v3.5.0) to analyze the pairwise correlation of proteins within each CORUM complex. The Spearman’s method was used to assess correlation of proteins within each complex. Subsequently, the Fisher z-transformation was performed to stabilize the variance of sample correlation coefficients in each condition, as described in [44, 45]. To avoid obtaining infinite z scores, all Spearman’s Rho values of 0.99 through 1 were replaced by 0.99 and those of − 1 through − 0.99 were replaced by − 0.99. For each CORUM complex, to determine whether the difference of mean z scores between two conditions (e.g., LG vs. N) is statistically significant, the following steps were performed: (1) 8 out of the 24 samples were randomly sampled twice and used as condition A and condition B, respectively; (2) for each condition, Spearman’s Rho values and z scores were computed as mentioned above; (3) the mean z score difference between conditions A and B was calculated; (4) the steps 1–3 were repeated for 10,000 times, and a null hypothesis distribution of the mean z score differences was generated; (5) the significance of each observed mean z score difference was computed using the null hypothesis distribution; and (6) the *p* values were adjusted by the Storey method for multiple comparison. CORUM protein complexes with *q* < 0.05 and mean z score differences of > 0.7 in absolute value were accepted as differentially regulated protein complexes. As the standard deviation of all random mean z score differences was 0.428, the cutoff for the mean z score difference of 0.70 corresponds to *p* < 0.1.

### Immunoblotting analysis

Immunoblotting analysis was performed essentially as we previously described [46, 47]. Ten micrograms of protein per sample was loaded onto mini-protean TGX gels (BIO-RAD, 4–20% gradient), separated by sodium dodecyl sulfate polyacrylamide gel electrophoresis (SDS-PAGE), and electro-transferred onto nitrocellulose membranes (BIO-RAD, 0.45 μm). Membranes were blocked with 5% nonfat dry milk (ApexBIO) in Tris-buffered saline containing 0.1% Tween 20 (TBST). All primary antibodies, including anti-XRCC5 (rabbit polyclonal antibody, #2753), anti-XRCC6 (rabbit monoclonal antibody, #4588), and GAPDH (rabbit monoclonal antibody, #3683), were purchased from Cell Signaling Technology (CST) and used at 1:1000 dilution in the aforementioned blocking buffer. After overnight incubation at 4 °C, membranes were washed with the TBST buffer and then incubated with 1:2000 diluted anti-rabbit secondary antibody (CST, #7074) for 1.5 h at room temperature. Protein bands were visualized following incubation with SuperSignal West Pico PLUS Chemiluminescent Substrate (Thermo Fisher Scientific) and exposure of membranes to autoradiography film (Thomas Scientific). Densitometry analysis was performed using ImageJ (v1.52a) on immunoblots from two independent immunoblotting experiments [48]. Pearson correlation analysis was conducted to calculate the correlation coefficient of protein expression levels.

## Results

### TMT-SPS-MS3 analysis for quantitative profiling of prostate tissue specimens

**Fig. 1:**
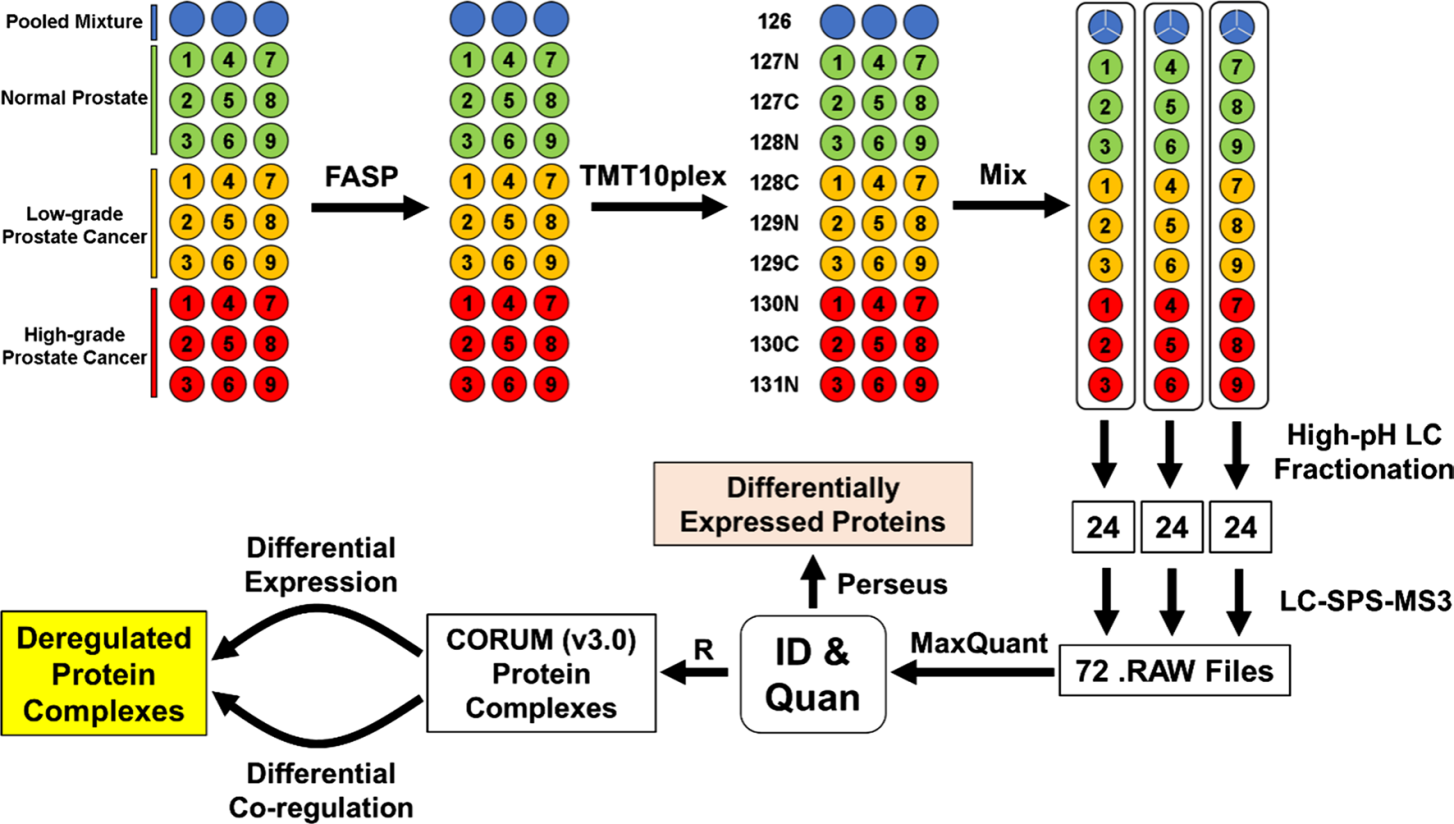
Workflow for quantitative proteomic comparison of three groups of prostate tissue specimens (i.e., normal control, low-grade PCa, and high-grade PCa) using TMT-SPS-MS3. A total of 30 OCT-embedded prostate tissue samples were digested in parallel into tryptic peptides by FASP, followed by chemical labeling with three sets of TMT10plex reagents. The three TMT126-labeled pooled mixture samples (shown as blue circles) were mixed and then equally divided into three portions (shown as blue circles divided into thirds). Differentially TMT10plex-labeled peptide samples were mixed into three groups (shown by the long rounded-rectangles), and then each set of TMT10plex mixture was fractionated by high-pH RPLC and concatenated into 24 fractions, so as to decrease peptide complexity and improve the detection of low-abundance proteins. Each fraction of TMT-labeled peptides was sequentially analyzed by LC-SPS-MS3. The acquired 72 RAW files derived from the three TMT10plex sets (24 fractions per set), which correspond to 30 individual samples, were analyzed by MaxQuant to identify and quantify proteins. Proteins quantified across all the 30 samples were a) analyzed by Perseus to identify differentially expressed proteins and b) mapped to CORUM (v3.0) protein complexes and subjected to differential expression and co-regulation analyses to identify in vivo deregulated protein complexes

The TMT-SPS-MS3 method was applied to quantitatively compare the proteomes across the 27 OCT-embedded prostate tissue samples, followed by differential expression and co-regulation analyses to identify in vivo deregulated protein complexes (Fig. 1). All tissue specimens had epithelial cell purity of ≥ 60% according to a histological analysis (Additional file 2: Table S1). The specimens were divided into three risk groups according to the prostatectomy Gleason scores, including PCa-adjacent normal prostate (abbreviated as N, n = 9), low-grade PCa (LG, n = 9), and high-grade PCa (HG, n = 9). Of note, the Gleason score (on a scale of 6 to 10) is one of the most commonly used systems for evaluating the aggressiveness of primary PCa, and a higher Gleason score is generally associated with a worse prognosis [49, 50]. Prior to protein quantification, the TMT labeling efficiency was assessed by a database searching analysis using TMT as a variable modification. Based on the ratios of peptide-spectrum matches (PSMs) for TMT-labeled peptides over total PSMs in different peptide fractions, the TMT labeling efficiency was determined as 98.7% ± 0.2% (mean ± SD) (Additional file 2: Table S2).

### Identification and analysis of DEPs

After TMT-SPS-MS3 analysis, database searching, and protein identification filtering, a total of 5562 protein groups were identified with an FDR of ≤ 1%, and on average 2585 (± 308) protein groups were identified from each peptide fraction (Additional file 2: Table S3). Among the 5562 identified protein groups, 5297, 4662, and 3642 were quantified in at least one, two, and three TMT10plex sets, respectively (Additional file 1: Fig. S1). The 3642 protein groups that were quantified across all the 27 samples were used for the following statistical and bioinformatic analyses.

**Fig. 2:**
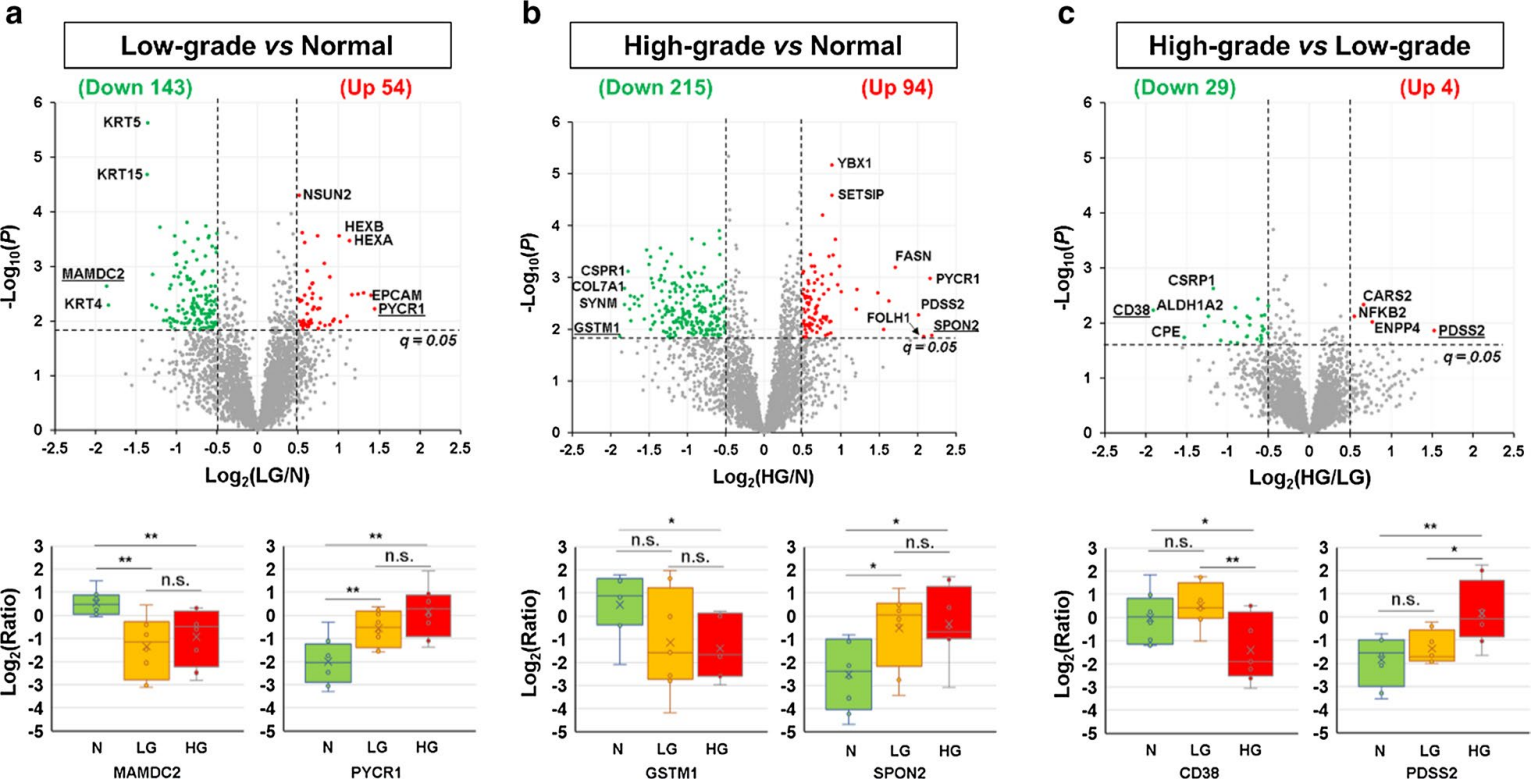
Identification of differentially expressed proteins between the N, LG, and HG groups. **a** Comparison of the N and LG groups. **b** Comparison of the N and HG groups. **c** Comparison of the LG and HG groups. The upper panels show the volcano plots of all the 3642 quantified protein groups. The lower panels show the boxplots for the most dramatically changed proteins in each comparison, whose names were underlined in the corresponding volcano plots. Here, the abbreviations N, LG, HG, and n.s. stand for normal prostate, low-grade PCa, high-grade PCa, and not significant, respectively. In the boxplots, **, *, and n.s. stand for *p* < 0.01, 0.01 ≤ *p* < 0.05, and *p* ≥ 0.05, respectively

To determine whether there were outlier samples, between-sample variation was assessed using the SuperHirn [37]. Three tissue samples (LG1, N5, and HG9) were found to have high degrees of between-sample variation (Additional file 1: Fig. S2), so they were considered as outlier samples and excluded from further statistical analysis. For the remaining 24 samples (n = 8 for each group), after Student’s *t* test (two-tailed) and multiple comparison correction, the cutoffs of *q* < 0.05 and log_2_-ratios of > 0.5 in absolute value were applied to identify DEPs (Additional file 2: Table S4). A total of 197 DEPs, including 143 downregulated and 54 upregulated proteins, were identified in LG samples in comparison to the N samples (Fig. 2a and Additional file 2: Table S5). Of these, MAM domain-containing protein 2 (MAMDC2) and pyrroline-5-carboxylate reductase 1 (PYCR1) were the most dramatically downregulated and upregulated proteins, respectively (Fig. 2a, lower panel). In HG samples (versus N samples), a total of 309 DEPs (215 downregulated and 94 upregulated) were identified, among which glutathione S-transferase mu 1 (GSTM1) and spondin-2 (SPON2) were the most dramatically downregulated and upregulated proteins, respectively (Fig. 2b and Additional file 2: Table S5). In comparison, the proteomic difference between LG and HG samples was small—only 33 DEPs (29 downregulated and 4 upregulated in HG, compared with LG) were identified, of which ADP-ribosyl cyclase/cyclic ADP-ribose hydrolase 1 (CD38) and decaprenyl diphosphate synthase subunit 2 (PDSS2) were the most substantially downregulated and upregulated proteins, respectively (Fig. 2c and Additional file 2: Table S6).

**Fig. 3:**
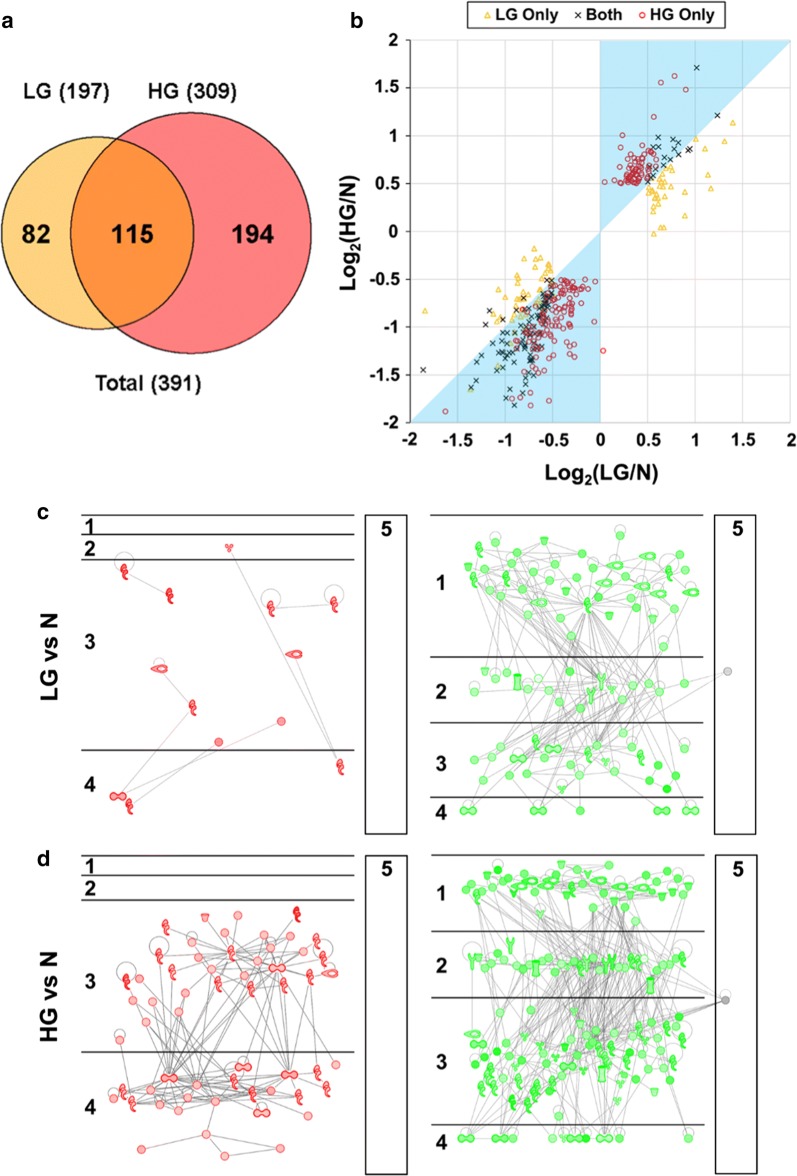
Comparison of protein groups differentially expressed in LG and HG PCa, compared with N samples. **a** Venn diagram of protein groups differentially expressed in LG and HG PCa, compared with N samples. A total of 82 DEPs were LG only, 194 were HG only, and 115 were shared by both LG and HG. **b** Scatter plot for the comparison of log_2_(LG/N) and log_2_(HG/N) ratios for the 82 LG-only, 115 shared, and 194 HG-only DEPs. The cyan shade covers the area where the absolute values of log_2_(LG/N) are less than those of log_2_(HG/N), i.e., the changes in the LG group are less remarkable than those in the HG group. **c** Putative networks of direct PPIs for proteins significantly upregulated (left) or downregulated (right) in LG PCa, compared with N samples. The five subcellular localization layers are (1) extracellular space, (2) plasma membrane, (3) cytoplasm, (4) nucleus, and (5) others. More detailed information is shown in Supplemental Figure S3. **d** Putative networks of direct PPIs for proteins significantly upregulated (left) or downregulated (right) in HG PCa, compared with N samples. The subcellular localization layers are the same for the panel C. More detailed information is shown in Supplemental Figure S4. In the figure, the abbreviations N, LG, and HG stand for normal prostate, low-grade PCa, and high-grade PCa, respectively

A comparison of the 197 DEPs in the LG (vs. N) group and the 309 DEPs in the HG (vs. N) group suggested that 115 DEPs are shared by the two groups, whereas 82 and 194 are unique to the LG and HG groups, respectively (Fig. 3a and Additional file 2: Table S5). Interestingly, most of the 194 DEPs unique to the HG group were also slightly (log_2_-ratios of − 0.5 to 0.5) changed in the LG group (Fig. 3b, red circles). Similarly, most of the 115 shared DEPs were less changed in the LG group than in the HG group (Fig. 3b, black “x”s). Collectively, the findings suggest that, compared with normal prostate, most protein expression level changes that are statistically significant in high-grade PCa were already present in low-grade PCa samples, although the extent is less pronounced. In contrast, most of the 82 DEPs unique to the LG group were less markedly changed in the HG group (Fig. 3b, orange triangles). Intriguingly, DAVID analysis of the 82 LG-only DEPs revealed a highly significant over-representation of extracellular exosomes (53 proteins, *p* = 2E − 24) (Additional file 2: Table S7), suggesting the possibility of differential exosome biogenesis and/or shedding in the two groups.

To determine whether the DEPs may interact with each other and form protein complexes, Ingenuity Pathway Analysis (IPA) was applied to reconstruct networks of proteins with direct PPI evidence that was experimentally obtained. In the LG group (vs. N), only 13 out of the 54 (24%) upregulated DEPs form three small PPI networks, but 92 out of the 143 (64%) downregulated DEPs form a large PPI network (Fig. 3c and Fig. S3). In comparison, in the HG group (vs. N), 58 out of the 94 (62%) upregulated DEPs form a large upregulated PPI network, and 157 out of the 215 (73%) downregulated DEPs form a large downregulated PPI network (Fig. 3d and Fig. S4). Notably, the upregulated protein subnetworks are almost exclusively localized in cytoplasm and nucleus (Fig. 3c and d; shown as compartments #3 and #4), whereas the downregulated protein subnetworks are mainly localized in extracellular space, plasma membrane, and cytoplasm (Fig. 3c, d; shown as compartments #1, 2, and 3).

### Identification of differentially expressed CORUM protein complexes

The CORUM database is a manually curated repository of experimentally characterized protein complexes from mammalian organisms, especially human (67%) [42, 51]. The latest CORUM database (v3.0) contains a total of 4274 protein complexes, representing one of the largest and most comprehensive publicly available datasets of mammalian protein complexes [42]. Notably, the CORUM database only comprises protein complexes that have been individually isolated and characterized. Therefore, it is generally considered a gold-standard database for mammalian protein complexes, being used in a large number of studies as a reference dataset for benchmarking computational models and high-throughput experimental data [52–54]. In comparison, although high-throughput interactome studies have identified many novel protein complexes, most of the novel identifications have not been corroborated by experiments delineating the biological function of complexes. Therefore, the high-quality CORUM database, rather than a high-throughput interactome database, was selected for the identification of deregulated protein complexes in this study.

**Fig. 4:**
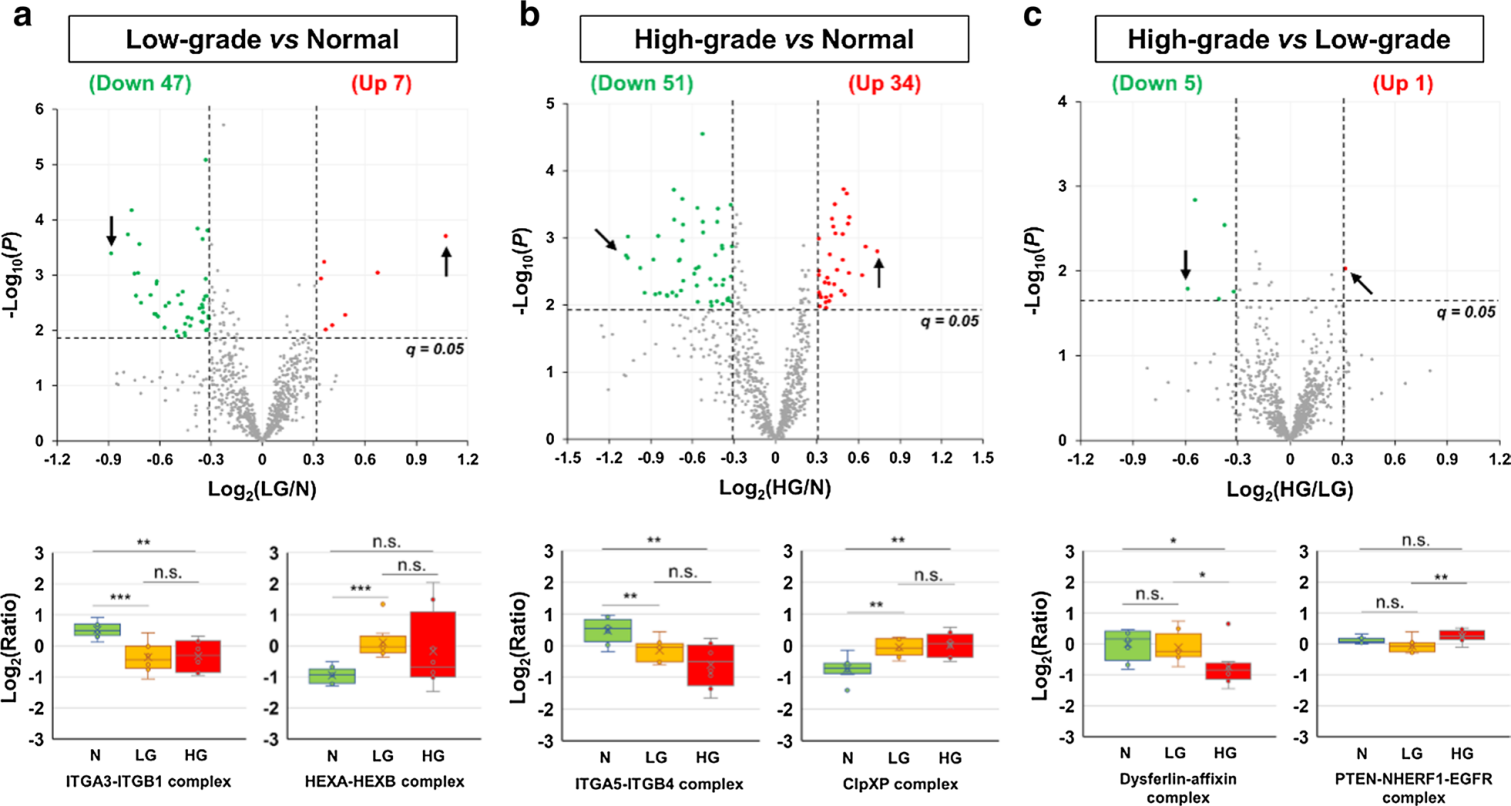
CORUM protein complexes differentially expressed between the N, LG, and HG groups. **a** Comparison of the N and LG groups. **b** Comparison of the N and HG groups. **c** Comparison of the LG and HG groups. The upper panels show the volcano plots of all the 852 quantified CORUM protein complexes. The lower panels show the boxplots for the most dramatically changed protein complexes in each comparison, which were indicated by arrows in the corresponding volcano plots. Here, the abbreviations N, LG, HG, n.s. stand for normal prostate, low-grade PCa, high-grade PCa, and not significant, respectively. In the boxplots, **, *, and n.s. stand for *p* < 0.01, 0.01 ≤ *p* < 0.05, and *p* ≥ 0.05, respectively

To identify specific protein complexes that are deregulated in primary PCa, the 3642 quantified protein groups were mapped to the CORUM (v3.0) database. A total of 852 non-redundant protein complexes, which met the criteria of containing at least two quantified subunits in each complex and having a subunit coverage of ≥ 50%, were selected for further analysis (Additional file 2: Table S8). To identify differentially expressed CORUM protein complexes, the log_2_-ratios of proteins in each complex were averaged for each tissue sample, followed by Student’s *t* test for the comparison between the three groups (i.e., N, LG, and HG). After applying the cutoffs of *q* < 0.05 and log_2_-ratios > 0.31 in absolute value, 54 (7 up and 47 down), 85 (34 up and 51 down), and 6 (1 up and 5 down) complexes were found to be differentially expressed in LG versus N, HG versus N, and HG versus LG comparisons, respectively (Fig. 4 and Additional file 2: Table S8). Among these, 27 (4 up and 23 down) complexes were differentially expressed in both cancer (LG and HG) groups, compared with the control (N) group (Additional file 1: Fig. S5 and Additional file 2: Table S8). In addition, 4 protein complexes were significantly less abundant in the HG group, compared with both N and LG groups (Additional file 1: Fig. S5B and Additional file 2: Table S8). Moreover, gene ontology (GO) enrichment analysis suggested that a) both the 47 complexes downmodulated in LG (vs. N) and the 51 complexes downmodulated in HG (vs. N) were significantly (*q* < 0.001) enriched in the GO term of cell adhesion (GO:0007155), to which 13 integrin protein complexes belong, and b) the 34 complexes overexpressed in HG (vs. N) were significantly (*q* = 0.03) enriched in the negative regulation of apoptotic process (GO:0043066), to which 4 Prothymosin alpha (ProTα) complexes belong (Additional file 2: Table S9).

### Identification of differentially regulated CORUM protein complexes

Protein–protein associations are essential for the assembly of functional protein complexes. Recent studies provided compelling evidence that co-regulation analysis of protein pairs, whose expression levels were quantified by isobaric tagging-based multiplexed quantitative proteomics approaches, permits the analysis of protein–protein associations with high accuracy [24, 43]. In the present study, to identify differentially associated/assembled protein complexes in vivo, differential co-regulation analysis was performed for a total of 475 non-redundant CORUM (v3.0) complexes meeting the criteria of (a) containing at least two quantified subunits, (b) having a subunit coverage of ≥ 50%, and (c) having a PPI coverage of ≥ 50%.

**Fig. 5:**
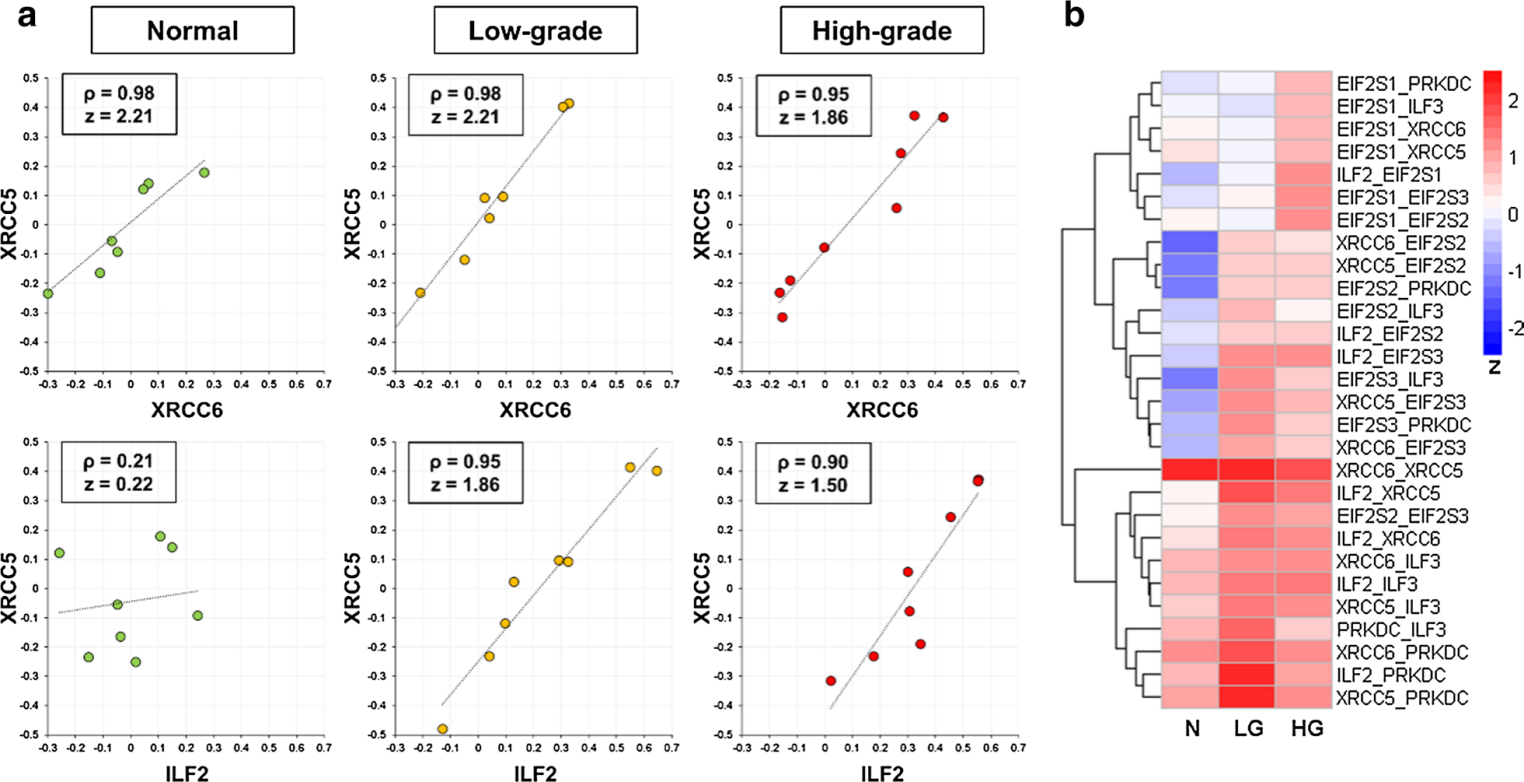
A representative example showing the differences of specific protein pairs in the DNA-PK-Ku-eIF2-NF90-NF45 protein complex across the three sample groups. **a** Scatter plots showing the log_2_-transformed relative abundance ratios of XRCC6 plotted against those of XRCC5 (upper panel) as well as the log_2_-transformed relative abundance ratios of ILF2 plotted against those of XRCC5 (lower panel) in the normal prostate, low-grade PCa, and high-grade PCa groups (from left to right). **b** Heatmap of z scores of all protein pairs (excluding self-pairs) within the DNA-PK-Ku-eIF2-NF90-NF45 protein complex. The abbreviations N, LG, and HG stand for normal prostate, low-grade PCa, and high-grade PCa, respectively

Firstly, for each of the 475 CORUM complexes, the Spearman correlation of each protein pair was computed and then converted into a z score to stabilize the variance [44, 45]. For instance, the DNA-PK-Ku-eIF2-NF90-NF45 complex, which plays a critical role in DNA double-strand break repair, is formed after the Ku heterodimer binds to a suitable DNA end [55, 56]. As expected, the XRCC5 and XRCC6 proteins—subunits of the stable Ku heterodimer [56] —have high Spearman’s Rho values and z scores in all the N, LG, and HG groups (Fig. 5a, upper panel). The high correlation coefficient of the protein expression levels of XRCC5 and XRCC6 was confirmed by an immunoblotting analysis, showing an average Pearson correlation coefficient of 0.89 (Additional file 1: Fig. S6). In comparison, the ILF2 and XRCC5 protein pair has low Spearman’s Rho and z score in the N group but has high values in the LG and HG groups (Fig. 5a, lower panel). This suggests increased protein–protein association between ILF2 and XRCC5 in the LG and HG groups, compared with the N group. A heatmap visualization of the z scores of all protein pairs within the complex showed that most protein pairs have higher z scores in the LG and HG groups than in the N group (Fig. 5b). It indicates that more DNA-PK-Ku-eIF2-NF90-NF45 complexes may be assembled in PCa cells than in normal prostate cells, probably in response to higher DNA damage in cancer cells.

**Fig. 6:**
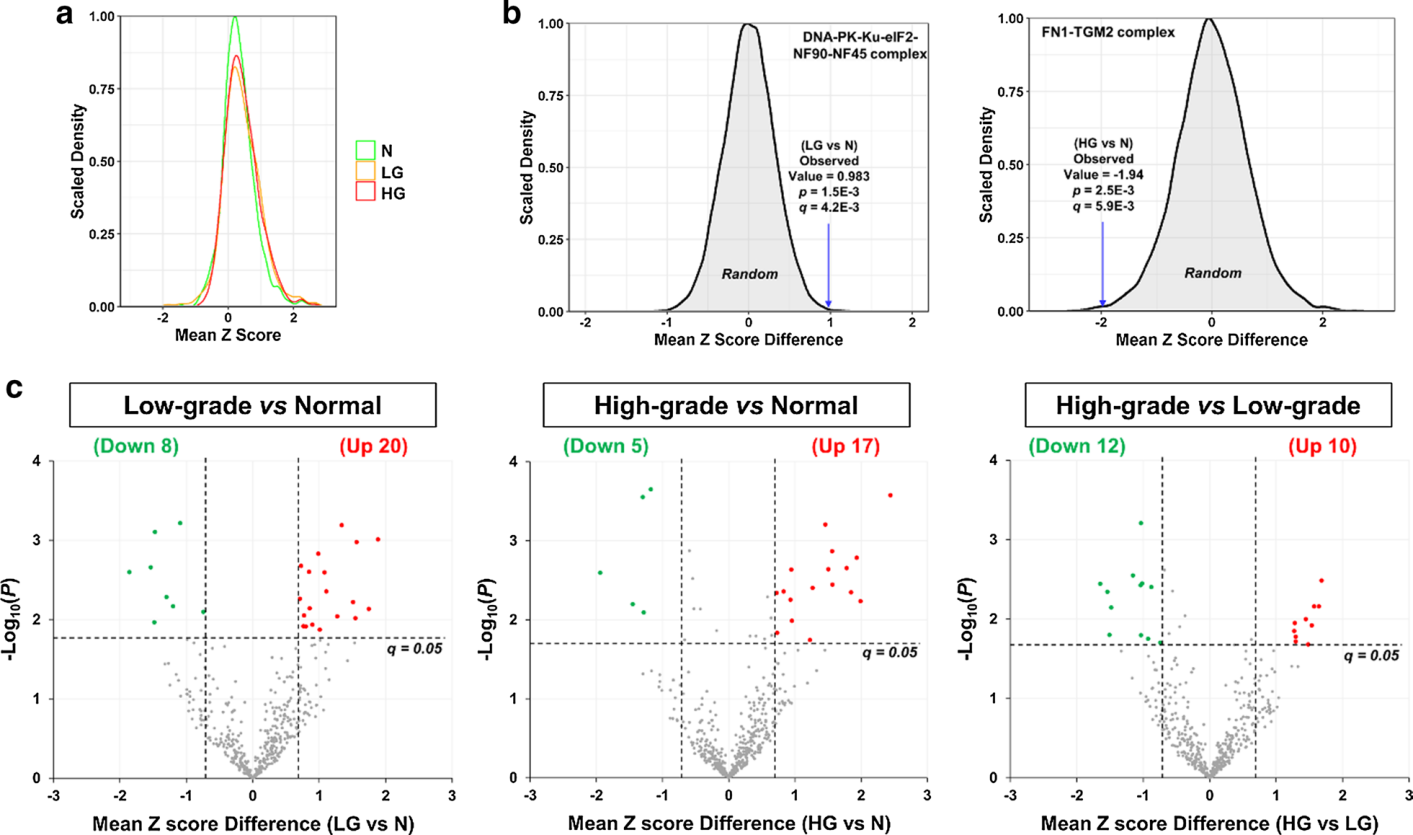
Identification of differentially associated CORUM protein complexes. The Fisher z-score transformation was performed on the Spearman’s rank-order correlation of proteins within each complex, in order to stabilize the variance of sample correlation coefficients. **a** Density plot of the mean z scores for the 475 CORUM protein complexes in N, LG, and HG samples. **b** Representative examples for the determination of *p* and *q* values corresponding to observed mean z score differences. Each peak shows the distribution of 10,000 mean z score differences between two sets of eight randomly selected samples. **c** Volcano plots showing mean z score differences plotted against negative log_10_-transformed *p* values for the 475 CORUM protein complexes. Here, the abbreviations N, LG, and HG stand for normal prostate, low-grade PCa, and high-grade PCa, respectively

Secondly, to statistically compare the assembly levels of each CORUM protein complex across the three groups, the z scores for all protein pairs within a complex were averaged and the mean z scores were used to estimate the assembly levels of the protein complexes. As shown in Fig. 6a, the mean z scores of all the 475 CORUM protein complexes in the N group roughly follow a normal distribution. In comparison, both the LG and HG groups have small shoulder peaks on the right side (Fig. 6a), suggesting that a small portion of the 475 protein complexes may have stronger protein–protein associations (i.e., higher assembly levels) in the LG and/or HG groups than in the N group.

Thirdly, for each of the 475 CORUM protein complexes, the distribution of 10,000 random mean z score differences was plotted, and the *p* value corresponding to an observed difference of mean z score between two groups was calculated. For example, for the DNA-PK-Ku-eIF2-NF90-NF45 complex upregulated in LG (vs. N), the 10,000 random mean z score differences follow a normal distribution (mean = 0, SD = 0.308). Therefore, the observed mean z score difference of 0.98 between the N and LG groups corresponds to *p* = 1.5E − 3 and, after multiple testing correction, *q* = 4.2E − 3 (Fig. 6b, left panel). For another example, for the FN1-TGM2 complex downregulated in HG (vs. N), the observed mean z score difference of -1.94 corresponds to *p* = 2.5E − 3 and, after multiple testing correction, *q* = 5.9E − 3 (Fig. 6b, right panel).

Finally, applying the cutoffs of *q* < 0.05 and mean z score difference of > 0.7 in absolute value, 28 (20 up and 8 down), 22 (17 up and 5 down), and 22 (10 up and 12 down) protein complexes were found to be differentially assembled in the LG versus N, HG versus N, and HG versus LG comparisons, respectively (Fig. 6c and Additional file 2: Table S10). Among these, only 4 (3 up and 1 down) complexes were differentially regulated in both cancer (LG and HG) groups, compared with the control (N) group (Additional file 1: Fig. S7 and Additional file 2: Table S10). Moreover, notably there is little overlap between the 58 differentially assembled protein complexes (Additional file 2: Table S10) and the 114 differentially expressed protein complexes (Additional file 2: Table S8)—only one protein complex was found to be differentially assembled and expressed in the same direction (i.e., up or down) and comparison (e.g., LG vs. N) (Additional file 1: Fig. S8). It suggests that differential co-regulation and expression analyses are highly complementary in detecting deregulated protein complexes.

GO enrichment analysis suggested that the 20 protein complexes upregulated in LG (vs. N) were significantly (*q* = 7.0E − 3) enriched in RNA splicing (GO:0008380), to which 6 protein complexes belong (Additional file 2: Table S11). In addition, at least 11 of the 20 protein complexes were annotated as being localized in nucleus (GO:0005634) (Additional file 2: Table S10), though the GO term enrichment did not reach significance after multiple testing correction (*p* = 9E − 4; *q *= 0.35). These nuclear protein complexes are involved in DNA damage response, chromatin remodeling, pre-mRNA and pri-miRNA processing, and RNA splicing (Additional file 1: Fig. S9). In addition, GO enrichment analysis also suggested that the 17 protein complexes upregulated in HG (vs. N) were significantly (*q* = 0.046) enriched in oxidative phosphorylation (GO:0006818), to which four subcomplexes of mitochondrial complex I belong (Additional file 2: Table S11).

## Discussion

### Proteins differentially expressed in PCa versus normal prostate tissue

In this study, nearly 400 proteins were found to be differentially expressed between PCa and normal prostate tissue. Notably, the total number of downregulated proteins is larger than that of upregulated proteins (Fig. 1). One concern is that the stromal cell ratios in PCa tissue samples were decreased compared with normal prostate tissue, a known phenomenon in the field of PCa research. Consequently, even when the cellular expression levels of stromal cell-enriched proteins are not changed, the total protein amount of such proteins may appear to be decreased, leading to the erroneous identification of these proteins as downregulated proteins. However, our histological analysis showed that the estimated average (± SD) stromal cell ratios (over all cells) were 32% (± 8%), 19% (± 8%), and 22 (± 7%) for the N, LG, and HG groups, respectively (Additional file 2: Table S1). Thus, although the stromal cell ratios were decreased in PCa samples compared to normal prostate, the largest decrease of average ratios is only 40% (i.e., 1–19/32). Furthermore, by applying the ESTIMATE (Estimation of Stromal and Immune Cells in Malignant Tumors using Expression data) algorithm to analyze the 3642 quantified proteins, the average stromal cell ratios were found to be 26% (± 8%), 17% (± 7%), and 20% (± 10%) for the N, LG, and HG groups, respectively (Additional file 2: Table S12), consistent with the histological analysis result. Moreover, of the 255 genes encoding the proteins downregulated in PCa samples (vs. normal prostate), only 18 (7% of 255) belong to the PCa stromal gene signature, which contains 105 genes significantly enriched (FDR < 1%; Log_2_FC > 1) in PCa stromal cells compared with PCa epithelial cells isolated by laser capture microdissection (Additional file 2: Table S13). Collectively, most of the protein downregulations were not directly caused by the decrease of stromal content in PCa compared with normal prostate.

Compared with normal prostate, the most substantially up- and down-regulated proteins in low-grade PCa were identified as PYCR1 and MAMDC2, respectively. PYCR1 is a metabolic enzyme that catalyzes the NAD(P)H-dependent conversion of pyrroline-5-carboxylate to proline [57]. Previous studies showed that (1) compared with normal prostate, PYCR1 was significantly upregulated in PCa at both mRNA and protein levels, (2) the expression levels of PYCR1 were significantly associated with Gleason scores, and 3) PYCR1 is involved in PCa cell proliferation and colony formation [58, 59]. MAMDC2 is a poorly characterized proteoglycan containing four MAM domains, which are commonly found in surface receptors [60]. To our knowledge, no other studies reported that MAMDC2 was substantially downregulated in low-grade PCa, compared with normal prostate.

Compared with normal prostate, the most dramatically up- and down-regulated proteins in high-grade PCa were identified as SPON2 and GSTM1, respectively. SPON2 is an extracellular matrix (ECM) protein belonging to the F-Spondin family. It was found to be a candidate serum and histological diagnostic biomarker for PCa and a candidate prognostic biomarker for colorectal cancer [61–64]. GSTM1 encodes a mu class cytoplasmic glutathione-*S*-transferase, which functions in cellular detoxification of many carcinogens. A recent meta-analysis suggested that GSTM1 deletion was significantly associated with risk of PCa in overall, Asian, Eurasian, and American populations [65].

In addition, a comparison of the DEPs in PCa (vs. normal) identified by us (n = 391) and by Iglesias-Gato et al. [11] (n = 649) suggested that 16 upregulated and 26 downregulated proteins were commonly found in both studies (Additional file 2: Table S5). The relatively small overlap is not unexpected because of the high heterogeneity of PCa and the differences of tissue preservation (OCT vs. FFPE), sample size (24 vs. 36), quantification method (TMT vs. Super-SILAC), and cutoff for identifying DEPs (*q *< 0.05 and |log2FC| > 0.5 vs. FDR < 0.1).

### Proteins differentially expressed in high-grade versus low-grade PCa

In contrast to the large number of DEPs between PCa and normal prostate, only 33 proteins were differentially expressed between low-grade and high-grade PCa specimens, even though the two groups have very different patient outcomes [49, 50]. With a larger sample size (n = 16 for high-risk PCa and n = 12 for low risk PCa), Iglesias-Gato et al. found that 130 proteins were differentially (*p *< 0.05 and FC > 1.6) expressed between low-risk and high-risk PCa groups [11]. Via label-free quantitative proteomics comparison of LCM-isolated epithelial cells from Gleason grade 3 versus 4 tumors (n = 4 for each), Staunton et al. identified 120 DEPs (FDR < 0.05) [12]. A comparison of the three datasets suggested that only three DEPs (ADH5, ALDH2, and CSRP1) are shared between our and Iglesias-Gato studies, three DEPs (CPE, QDPR, and PACSIN3) are shared between our and Staunton studies, and five DEPs (ABAT, COL6A2, COL6A3, EPHX2, and PFKP) are shared between the Iglesias-Gato and Staunton studies (Additional file 2: Table S6). The small overlaps between the three studies are likely due to high PCa heterogeneity as well as differences of tissue preservation methods, sample sizes, quantification methods, and cutoffs for identifying DEPs.

In this study, the most notably up- and down-regulated proteins in high-grade PCa compared with low-grade PCa were PDSS2 and CD38, respectively. PDSS2, an enzyme that synthesizes the prenyl side-chain of coenzyme Q, was included in the Promark panel for the prediction of PCa aggressiveness and lethality [66, 67]. CD38, a cyclic ADP-ribose synthase, is the main NAD’ase in cells. Recent studies indicated that (1) decreased expression of CD38 in luminal progenitor cells can initiate PCa and is linked to lower overall survival, (2) CD38 expression inversely correlates with PCa progression, and 3) CD38 inhibits PCa proliferation by reducing cellular NAD^+^ pools [68, 69].

### The putative PPI networks of DEPs

The present study revealed that, compared with normal prostate, both low-grade and high-grade PCa have decreased expression of many ECM and plasma membrane proteins, which form large putative PPI networks. The ECM remodeling is probably due to increased proteolysis by matrix metalloproteinases, including contributions from extracellular vesicles [70]. The most striking difference between low-grade and high-grade PCa is that only small PPI networks were more abundant in the former group, whereas a large PPI network was upregulated in the latter group. This suggests that high-grade PCa cells may potentially perturb protein complexes more broadly than low-grade PCa as a feature and/or driver of higher aggressiveness.

### Differentially expressed CORUM protein complexes

The GO enrichment analysis of differentially expressed CORUM protein complexes revealed cell adhesion (GO:0007155) as the most prominently downregulated process in both low-grade and high-grade PCa, compared to normal prostate (Additional file 2: Table S9). Of note, all the downregulated protein complexes belonging to this GO term (cell adhesion) are integrin protein complexes. Integrins are cell surface receptors for extracellular matrix proteins and play important roles in cell survival, proliferation, and migration [71]. Previous studies demonstrated that many integrin subunits were downregulated in PCa [71]. In addition, the negative regulation of apoptotic process (GO:0043066), involving four ProTα complexes, was found to be significantly upregulated in high-grade PCa compared with normal prostate (Additional file 2: Table S9). ProTα is a 109–111 amino acid protein that acts as an anti-apoptotic factor involved in the control of the apoptosome activity in the cytoplasm [72]. The expression of ProTα was found to be positively correlated with the development and progression of human PCa as well as many other cancer types such as breast, colon, liver, and lung cancers [73]. Nevertheless, the functions of ProTα and its protein complexes in PCa remain to be better defined.

### Differentially regulated protein complexes

The combination of protein co-regulation analysis and GO enrichment analysis revealed that six spliceosome subcomplexes related to RNA splicing (GO:0008380) were significantly increased in low-grade PCa, compared with normal prostate (Additional file 2: Table S11). RNA alternative splicing allows for the production of multiple proteins from a single gene, thereby expanding protein diversity. Accumulating studies have shown that the alternative splicing of key cellular regulatory proteins, such as fibroblast growth factor receptor, androgen receptor, cyclin D1, and Kruppel-like factor 6, contributes to the tumorigenesis and progression of PCa [74]. Although beyond the scope of this study, we believe it would be valuable to confirm the increased assembly of spliceosomes in early stage PCa and to better define the roles of spliceosomes in PCa tumorigenesis.

The most prominent complexes with higher assembly levels in high-grade PCa, compared with normal prostate, are subcomplexes of mitochondrial complex I (Additional file 2: Table S11). As the largest complex of the mitochondrial electron transport chain, mitochondrial complex I contributes ~ 40% of the proton motive force required for mitochondrial ATP synthesis [75]. Moreover, via modulating the NAD +/NADH ratio, mitochondrial complex I controls the synthesis of aspartate, a precursor of purine and pyrimidine synthesis. Although still controversial, epidemiological studies demonstrated that Metformin, a mitochondrial complex I inhibitor, reduces incidence and mortality of PCa patients [76]. It would be interesting to test whether PCa patients with different assembly levels of mitochondrial complex I in PCa cells may benefit differently from Metformin treatment.

A major challenge for the management of PCa patients is to distinguish aggressive from indolent PCa. In this study, 22 protein complexes were found to be differentially assembled in high-grade (high-risk) PCa compared with low-grade (low-risk) PCa. Among most dramatically dysregulated protein complexes, the GRB2-SOS1 complex has significantly higher (Z score difference = 1.57, *q* = 0.017) assembly level, whereas the PPP2CA-PPP2R1A complex has significantly lower (Z score difference = − 1.64, *q* = 0.009) assembly level, in high-grade PCa than in low-grade PCa. The GRB2-SOS1 complex is a key component of the receptor tyrosine kinase-Ras signaling pathway whose hyper-activation drives PCa progression [77]. The PPP2CA-PPP2R1A complex is a critical component of protein phosphatase 2A, which dephosphorylates a large number of oncogenic proteins and thus inhibits multiple oncogenic signaling pathways [78]. It is possible that increased assembly of GRB2-SOS1 complex and disruption of PPP2CA-PPP2R1A complex may play a synergistic role in promoting PCa aggressiveness.

Until now, it has only been convincingly demonstrated that isobaric labeling (e.g., TMT and iTRAQ)-based quantification allows the identification of protein–protein associations with high accuracy through protein co-regulation analysis [24, 43]. Compared with label-based quantification, label-free quantification (LFQ) is more straightforward and scalable. Notably, the past few years have witnessed significant improvement of quantification accuracy of LFQ methods [79, 80]. It would be interesting and valuable to explore whether LFQ quantification followed by protein co-regulation analysis can also identify deregulated protein–protein associations with high accuracy.

## Limitations

Similar to all other comprehensive proteomic studies of prostate tissue specimens [7, 11–15], the sample size in this study is relatively small. In addition, other categories of prostate tissue specimens, such as those of benign prostatic hyperplasia, PCa with Gleason scores of 7, and more importantly metastatic PCa, were not investigated. Nonetheless, larger-scale analysis of prostate tissue specimens in the near future will clarify the landscape of global protein complex changes during PCa development and progression, facilitating the discovery of novel biomarkers and drug targets for precision management of PCa patients.

In addition, due to the wide dynamic range of protein abundance in tissue specimens, the present proteomic dataset is biased towards high-abundance protein complexes. Thus, the differences of many low-abundance protein complexes between the three groups (i.e., N, LG, and HG) could not be compared. However, with continuous improvements in proteomics instrumentation and methods, near-complete analysis of protein complexes will be achieved in the near future.

## Conclusions

In summary, TMT-SPS-MS3 profiling of clinical prostate tissue specimens, followed by differential expression and co-regulation analyses, led to the discovery of candidate dysregulated protein complexes in primary PCa (Additional file 2: Table 1). Compared with normal prostate, low-grade PCa tissue samples have (1) seven more abundant protein complexes, (2) higher assembly levels of 20 protein complexes including six spliceosome subcomplexes involved in RNA splicing, (3) decreased abundance of 47 protein complexes including 13 integrin protein complexes involved in cell adhesion, and (4) decreased assembly levels of eight protein complexes. Compared with normal prostate, high-grade PCa tissue samples have (1) 34 more abundant protein complexes including four anti-apoptotic ProTα complexes, (2) increased assembly of 17 complexes including four subcomplexes of mitochondrial complex I, (3) 51 less abundant complexes including 13 integrin complexes involved in cell adhesion, and (4) decreased assembly of five complexes. In addition, compared with low-grade PCa, high-grade PCa tissue samples have (1) one more abundant protein complex, (2) increased assembly of 10 protein complexes including the GRB2-SOS1 complex, (3) five less abundant protein complexes, and (4) decreased assembly of 12 complexes including the PPP2CA-PPP2R1A complex. To our knowledge, this study represents the first comprehensive, albeit indirect, analysis of individual protein complexes in PCa tissue specimens. It may serve as a useful resource for better understanding the dysregulation of protein complexes in primary PCa.

**Table 1:** Summary of the results for differential expression and co-regulation analyses

Group	Category	Cutoffs for filtering	LG versus N	HG versus N	HG versus LG
Individual proteins	Total	Quantified in all samples	3642	3642	3642
	Differential	q < 0.05; |Log_2_FC| > 0.5	197	309	33
	Up	q < 0.05; Log_2_FC > 0.5	54^a^	94^b^	4
	Down	q < 0.05; Log_2_FC < − 0.5	143^c^	215^c^	29
CORUM complexes for differential expression analysis	Total	Quantified subunits ≥ 2; subunit coverage ≥ 50%	852	852	852
	Differential	q < 0.05; |Log_2_FC| > 0.31	54	85	6
	Up	q < 0.05; log_2_FC > 0.31	7	34^d^	1
	Down	q < 0.05; Log_2_FC < − 0.31	47^e^	51^e^	5
CORUM complexes for differential co-regulation analysis	Total	Quantified subunits ≥ 2; subunit coverage ≥ 50%; PPI coverage ≥ 50%	475	475	475
	Differential	q < 0.05; |mean Z score difference| > 0.7	28	22	22
	Up	q < 0.05; mean Z score difference > 0.7	20^f^	17^g^	10
	Down	q < 0.05; mean Z score difference < − 0.7	8	5	12

^a^Form three small PPI networks

^b^Form a large PPI network in cytoplasm and nucleus

^c^Form a large PPI network mainly in extracellular space, plasma membrane, and cytoplasm

^d^Four ProTα complexes are involved in the negative regulation of apoptotic process (GO:0043066)

^e^Thirteen integrin protein complexes are involved in cell adhesion (GO:0007155)

^f^Eleven protein complexes are nuclear complexes, of which six are involved in RNA splicing (GO:0008380)

^g^Four subcomplexes of mitochondrial complex I are involved in oxidative phosphorylation (GO:0006818)

## Additional files

**Additional file 1.** Supplemental figures S1–S9.

**Additional file 2.** Supplemental tables S1–S13.

## Abbreviations

DAVID: the database for annotation, visualization and integrated discovery
DEP: differentially expressed proteins
ECM: extracellular matrix
ESTIMATE: estimation of stromal and immune cells in malignant tumors using expression data
FASP: filter-aided sample preparation
FDR: false discovery rate
FFPE: formalin-fixed and paraffin-embedded
GO: gene ontology
H and E: hematoxylin and eosin
HG: high-grade
IPA: ingenuity pathway analysis
LC: liquid chromatography
LCM: laser capture microdissection
LFQ: label-free quantification
LG: low-grade
MALDI: matrix-assisted laser desorption/ionization
MS: mass spectrometry
N: normal
OCT: optimal cutting temperature
PCa: prostate cancer
ProTα: prothymosin alpha
PPI: protein-protein interaction
RPLC: reverse-phase liquid chromatography
SD: standard deviation
SPS: synchronous precursor selection
2DE: two-dimensional electrophoresis
TMT: tandem mass tag
