# Supplementary material for: Quantitative proteomic analysis of prostate tissue specimens identifies deregulated protein complexes in primary prostate cancer
Source: Clin Proteomics. 2019 Apr 13;16:15. doi: 10.1186/s12014-019-9236-2 (PMC6461817; doi:10.1186/s12014-019-9236-2)
Supplement: Supplementary file 1 — Additional file 1. Supplemental figures S1–S9. [file 12014_2019_9236_MOESM1_ESM.docx]

**Supplementary Figures for**

**Quantitative Proteomic Analysis of Prostate Tissue Specimens Identifies Deregulated Protein Complexes in Primary Prostate Cancer**

Bo Zhou, Yiwu Yan, Yang Wang, Sungyong You, Michael R. Freeman, Wei Yang^*^

Division of Cancer Biology and Therapeutics, Departments of Surgery and Biomedical Sciences, Samuel Oschin Comprehensive Cancer Institute, Cedars-Sinai Medical Center, 8700 Beverly Boulevard, Los Angeles, CA 90048

^*^Corresponding Author

Rm. 4009, Davis Research Bldg

Cedars-Sinai Medical Center

8700 Beverly Blvd

Los Angeles, CA 90048

Email: [wei.yang@cshs.org](mailto:wei.yang@cshs.org)

Phone: +1 (310)423-7142


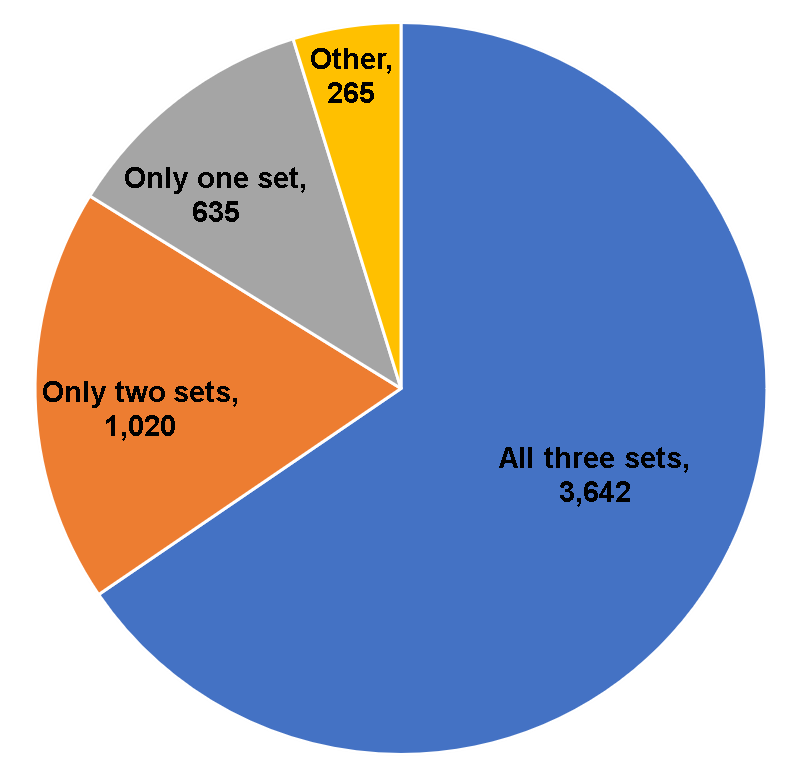


**Figure S1. Pie chart of protein groups quantified in different TMT10plex sets.** Of all the 5,562 identified protein groups, 3,642 protein groups were quantified in all the three TMT10plex sets (30 samples), 4,662 (3,642 + 1,020) protein groups were quantified in at least two TMT10plex sets, and 5,297 (4,662 + 635) protein groups were quantified in at least one TMT10plex set.


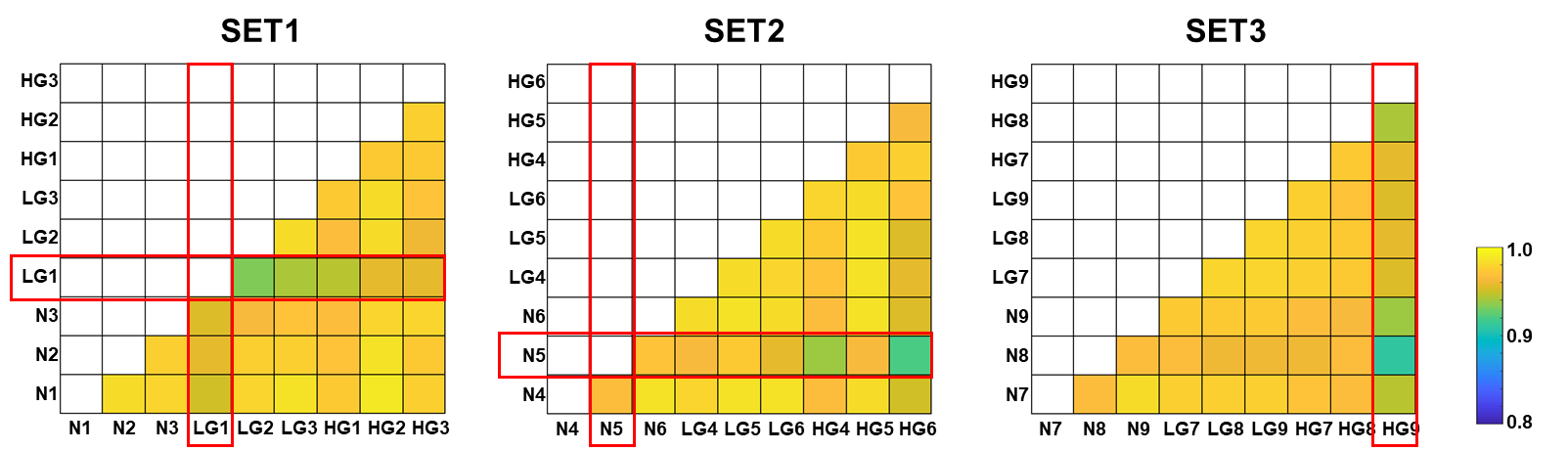


**Figure S2. Detection and removal of outlier samples.** The outlier samples were detected in each TMT10plex set using the SuperHirn, followed by visual inspection. The samples LG1, N5, and HG9 have poor similarity patterns with other samples in the same TMT set (shown in long red boxes). Hence, they were considered as outlier samples and removed prior to further analysis.


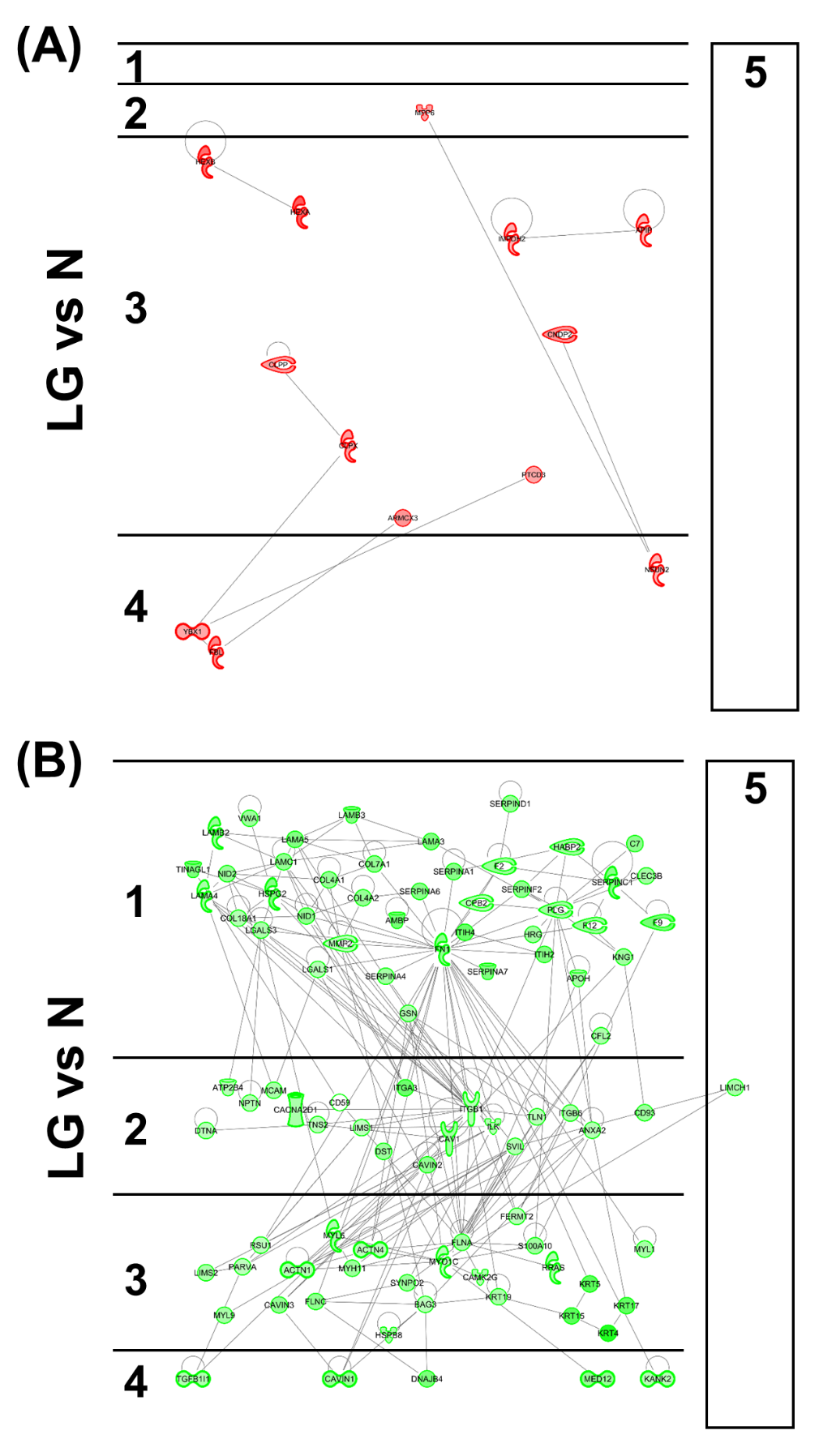


**Figure S3. Putative networks of direct PPIs for proteins significantly (A) upregulated or (B) downregulated in low-grade (LG) PCa, compared with PCa-adjacent normal (N) controls.** The five subcellular localization layers are 1) extracellular space, 2) plasma membrane, 3) cytoplasm, 4) nucleus, and 5) others.


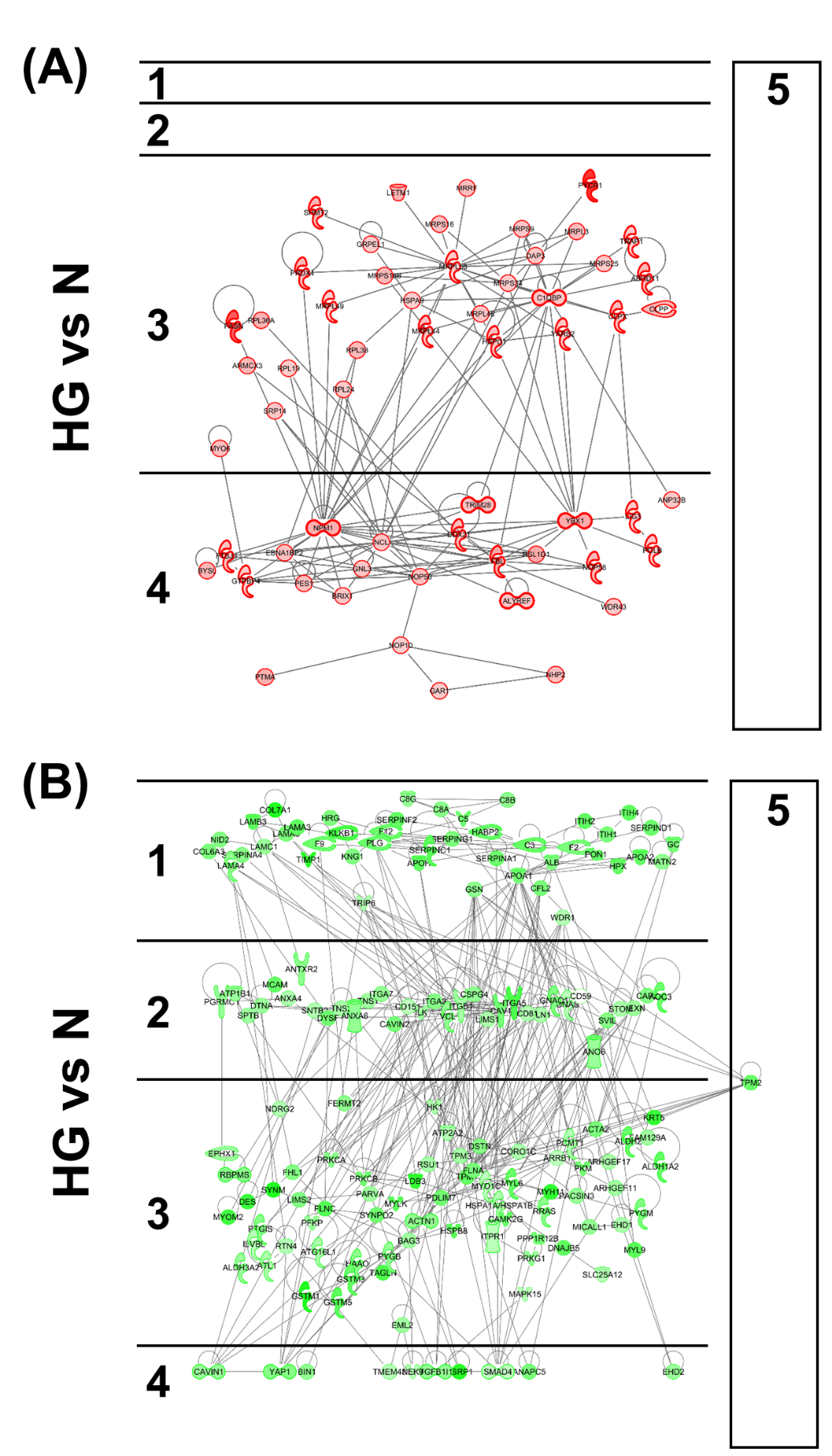


**Figure S4. Putative networks of direct PPIs for proteins significantly (A) upregulated or (B) downregulated in high-grade (HG) PCa, compared with PCa-adjacent normal (N) controls.** The five subcellular localization layers are 1) extracellular space, 2) plasma membrane, 3) cytoplasm, 4) nucleus, and 5) others.


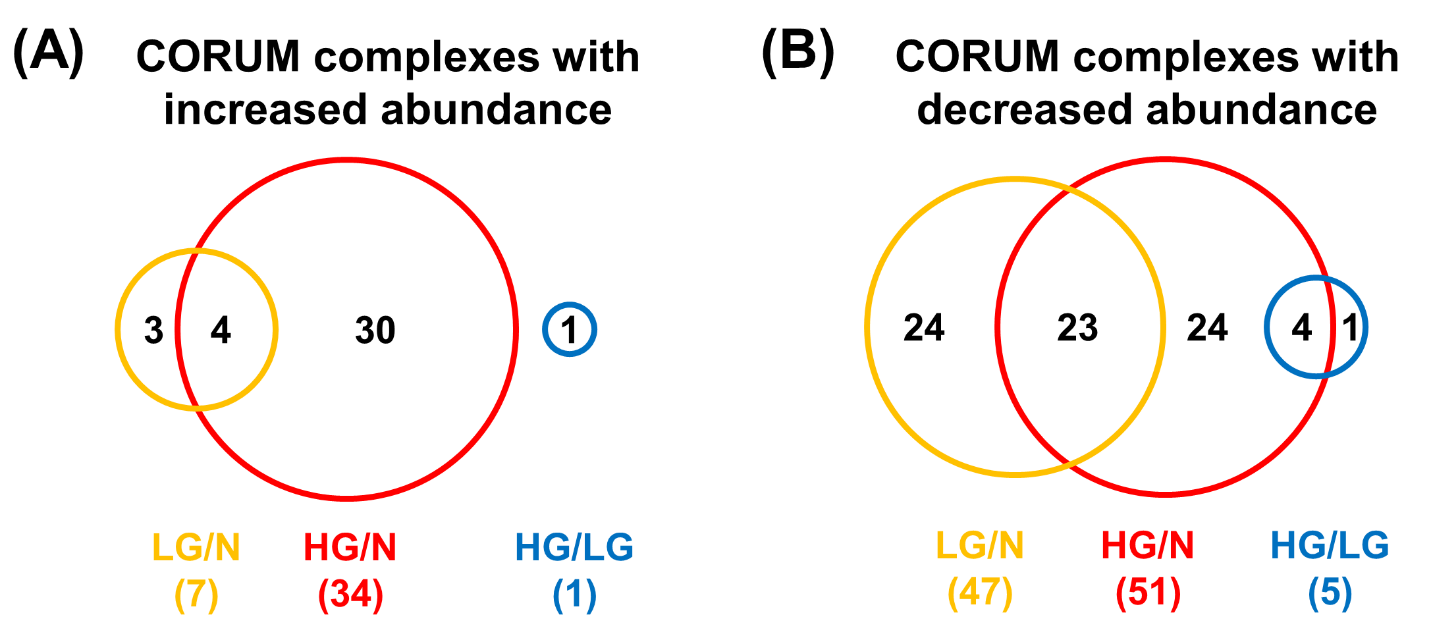


**Figure S5. Venn diagrams of differentially expressed protein complexes.** **(A)** A total of 7, 34, and 1 CORUM protein complexes were more abundant in LG (vs N), HG (vs N), and HG (vs LG), respectively. Of these, 4 protein complexes were more abundant in both LG and HG groups, compared with the N group. **(B)** A total of 47, 51, and 5 CORUM protein complexes were less abundant in LG (vs N), HG (vs N), and HG (vs LG), respectively. Of these, 23 protein complexes were less abundant in both LG and HG groups, compared with the N group. In addition, compared with both N and LG groups, 4 protein complexes were less abundant in the HG group. Here, the abbreviations N, LG, and HG stand for normal prostate, low-grade prostate cancer, and high-grade prostate cancer, respectively.


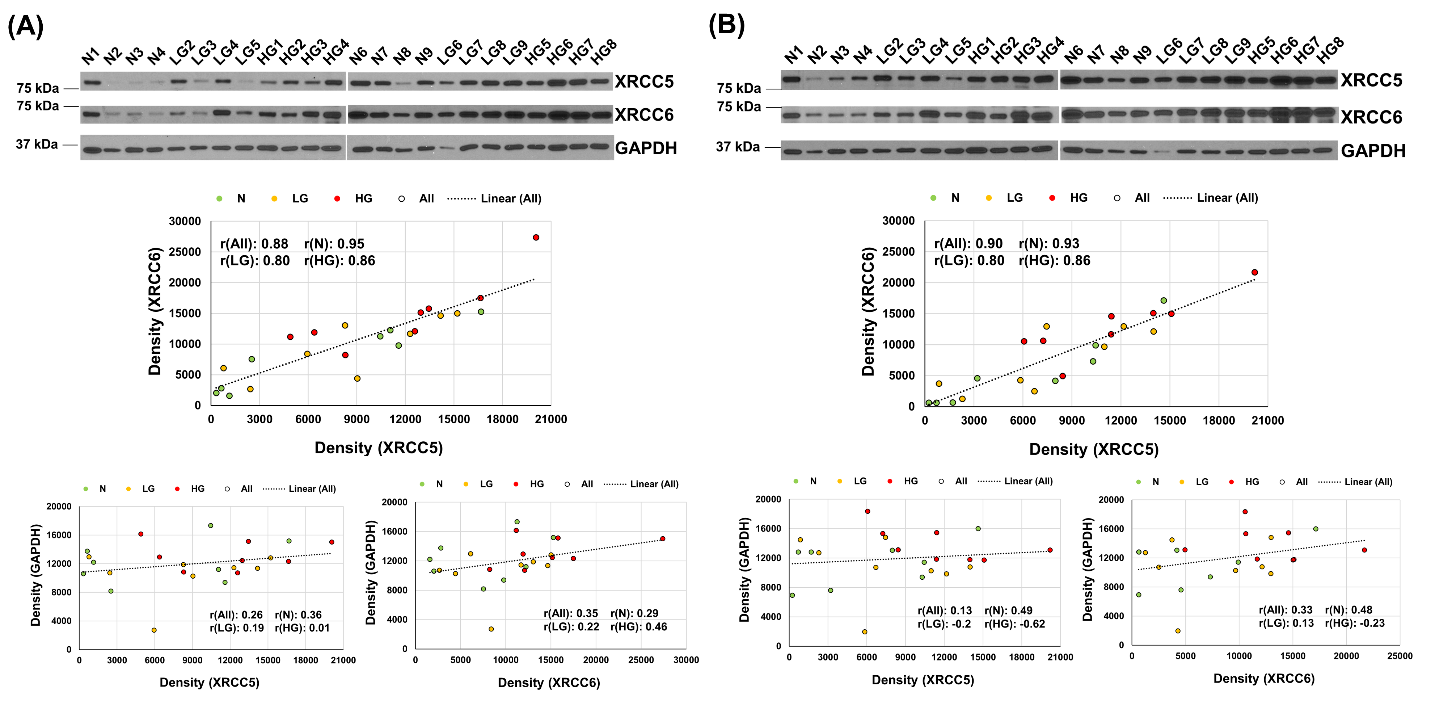


**Figure S6. Immunoblotting validation of the strong co-expression of XRCC5 and XRCC6 proteins in prostate cancer tissue samples.** (A) and (B) show the results of the first and second independent replicates of the immunoblotting experiments, respectively. XRCC5 and XRCC6, which form stable heterodimers in nuclei, have strong (*r* = 0.89 on average) correlation at protein expression levels. In contrast, both XRCC5 and XRCC6 proteins have weak (*r* = 0.30 and *r* = 0.23 on average, respectively) correlation at protein expression levels with GAPDH, a cytoplasmic protein. Because each gel can only accommodate up to 15 samples, the 24 tissue lysate samples were divided into two groups (12 samples per group), for which the SDS-PAGE separation and immunoblotting were performed in parallel.


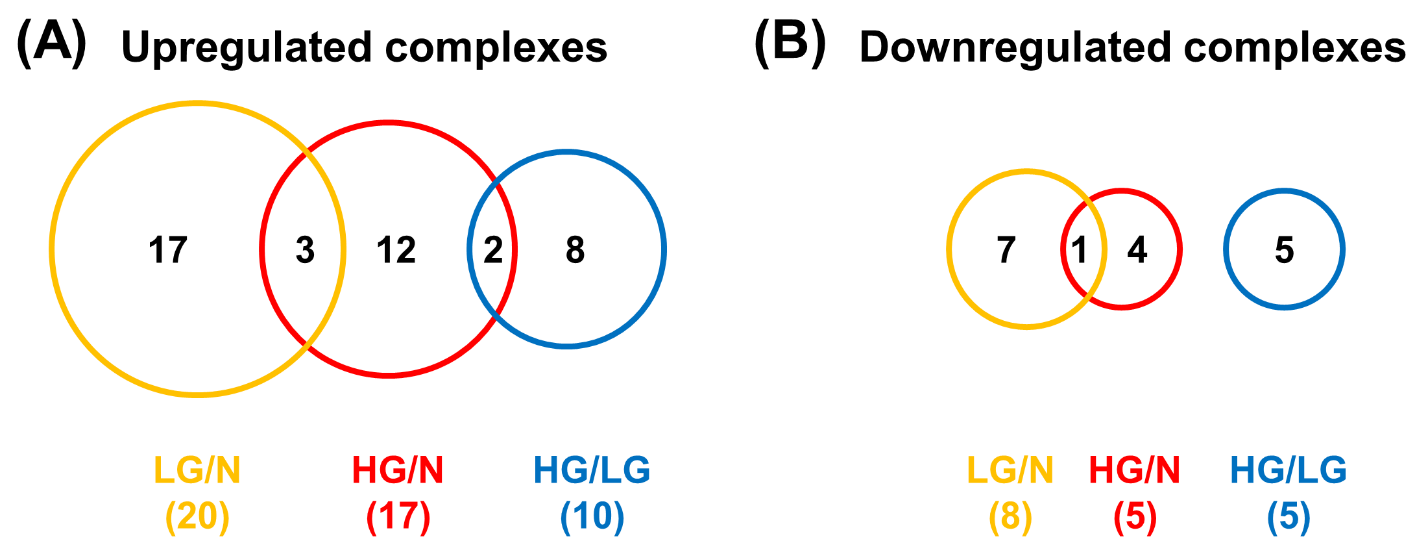


**Figure S7. Venn Diagram of differentially co-regulated protein complexes.** **(A)** A total of 20, 17, and 10 CORUM protein complexes were upregulated in LG (vs N), HG (vs N), and HG (vs LG), respectively. Of these, 3 protein complexes were upregulated in both prostate cancer (LG and HG) groups, compared with the N group. In addition, 2 protein complexes were upregulated in the HG group, compared with the N and LG groups. **(B)** A total of 8, 5, and 5 CORUM protein complexes were downregulated in LG (vs N), HG (vs N), and HG (vs LG), respectively. Of these, only one protein complex was downregulated in both prostate cancer (LG and HG) groups, compared with the N group. Here, the abbreviations N, LG, and HG stand for normal prostate, low-grade prostate cancer, and high-grade prostate cancer, respectively.


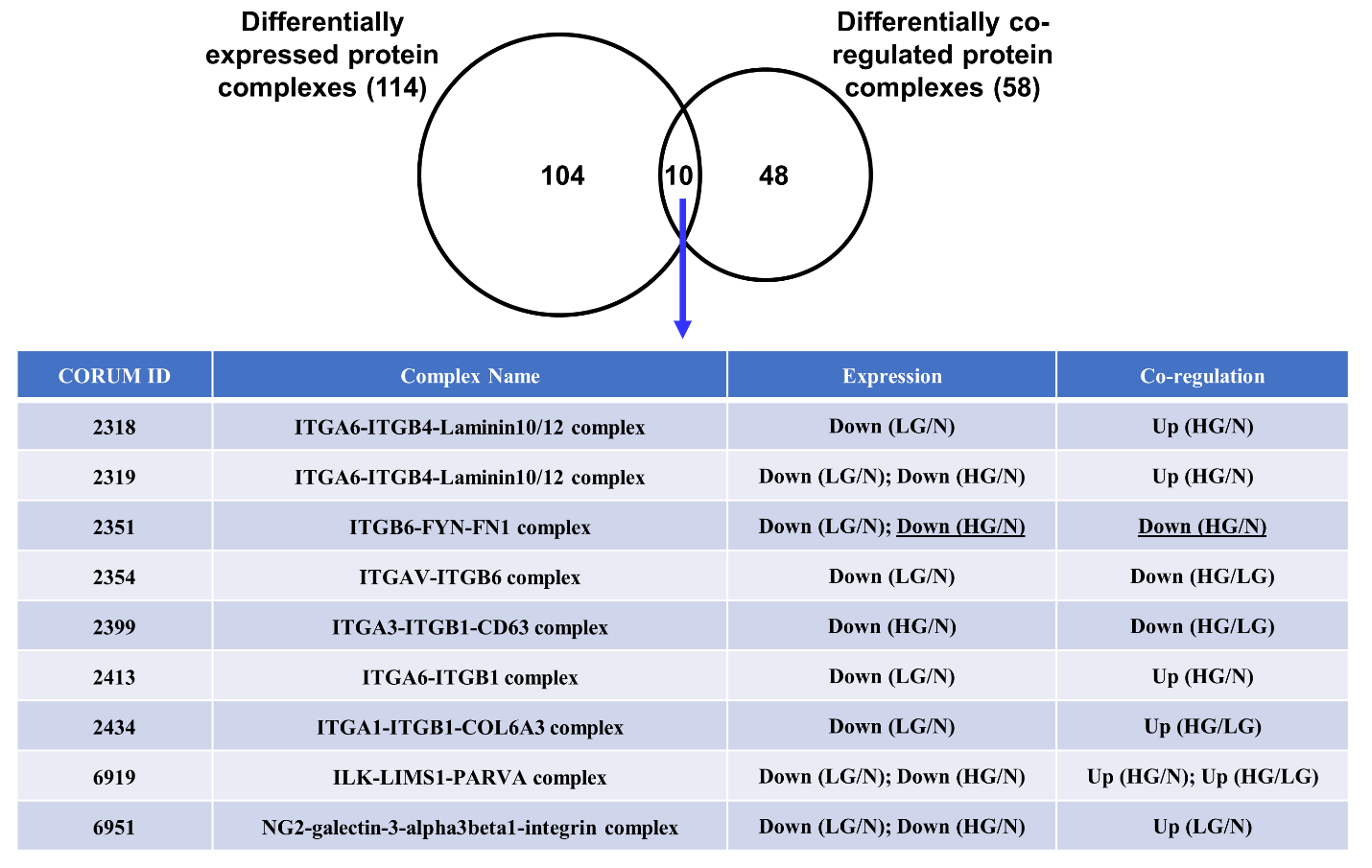


**Figure S8. Essentially all differentially associated/assembled protein complexes are not significantly differentially expressed.** The 114 differentially expressed protein complexes and the 58 differentially assembled protein complexes only share 10 overlapping complexes. Of these, however, only one protein complex (the ITGB6-FYN-FN1 complex, underlined in the table) is downregulated at both expression and assembly (co-regulation) levels in the sample comparison (high-grade prostate cancer vs normal prostate).


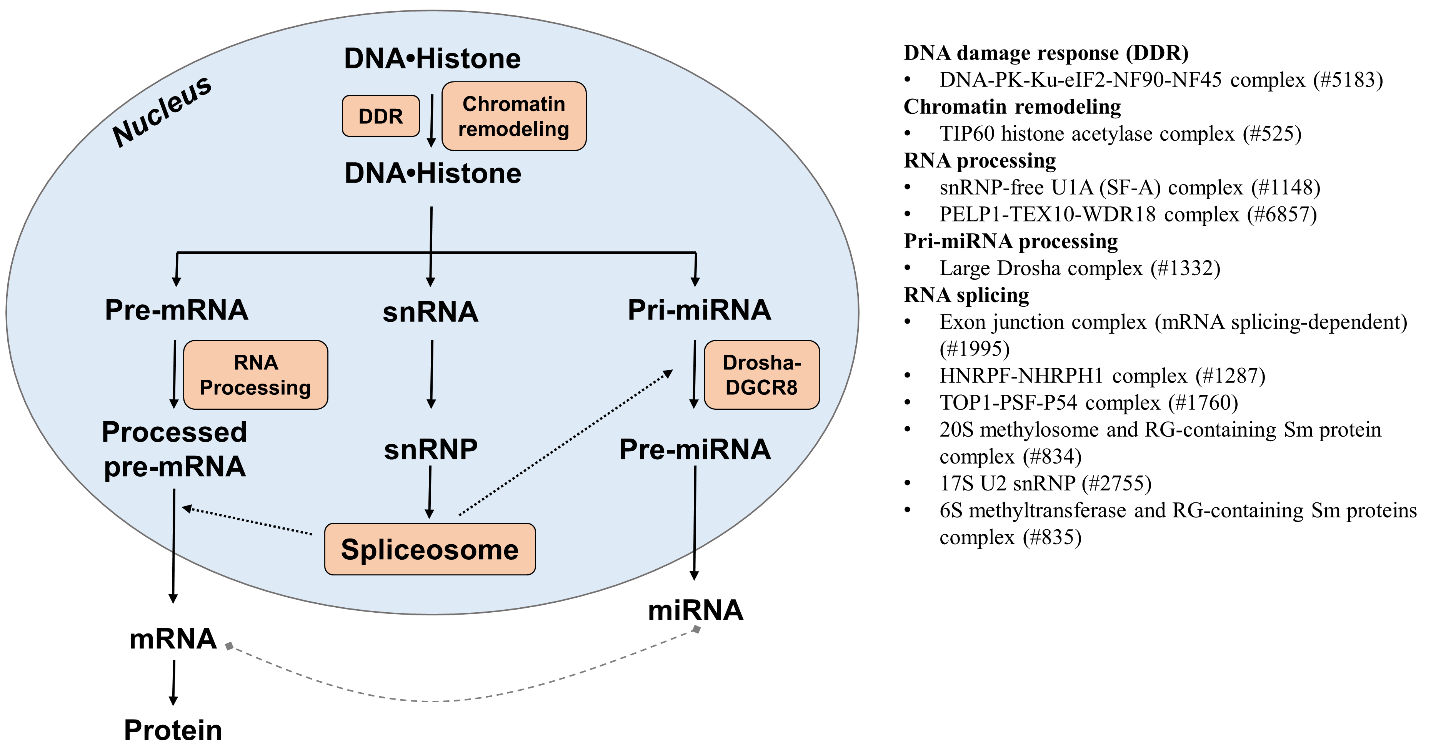


**Figure S9. Biological processes involving the nuclear protein complexes with increased assembly levels in low-grade prostate cancer, compared with normal prostate.** The numbers in parentheses represent the CORUM complex ID numbers.
